# Reliability of plasma polar metabolite concentrations in a large-scale cohort study using capillary electrophoresis-mass spectrometry

**DOI:** 10.1371/journal.pone.0191230

**Published:** 2018-01-18

**Authors:** Sei Harada, Akiyoshi Hirayama, Queenie Chan, Ayako Kurihara, Kota Fukai, Miho Iida, Suzuka Kato, Daisuke Sugiyama, Kazuyo Kuwabara, Ayano Takeuchi, Miki Akiyama, Tomonori Okamura, Timothy M. D. Ebbels, Paul Elliott, Masaru Tomita, Asako Sato, Chizuru Suzuki, Masahiro Sugimoto, Tomoyoshi Soga, Toru Takebayashi

**Affiliations:** 1 Department of Preventive Medicine and Public Health, Keio University School of Medicine, Tokyo, Japan; 2 Institute for Advanced Biosciences, Keio University, Tsuruoka, Yamagata, Japan; 3 Department of Epidemiology and Biostatistics, School of Public Health, Faculty of Medicine, Imperial College London, London, United Kingdom; 4 MRC-PHE Centre for Environment and Health, Imperial College London, London, United Kingdom; 5 Department of Obstetrics and Gynecology, Keio University School of Medicine, Tokyo, Japan; 6 Faculty of Environment and Information Studies, Keio University, Fujisawa, Kanagawa, Japan; 7 Computational and Systems Medicine, Department of Surgery and Cancer, Imperial College London, South Kensington, London, United Kingdom; National Research Council of Italy, ITALY

## Abstract

**Background:**

Cohort studies with metabolomics data are becoming more widespread, however, large-scale studies involving 10,000s of participants are still limited, especially in Asian populations. Therefore, we started the Tsuruoka Metabolomics Cohort Study enrolling 11,002 community-dwelling adults in Japan, and using capillary electrophoresis-mass spectrometry (CE-MS) and liquid chromatography–mass spectrometry. The CE-MS method is highly amenable to absolute quantification of polar metabolites, however, its reliability for large-scale measurement is unclear. The aim of this study is to examine reproducibility and validity of large-scale CE-MS measurements. In addition, the study presents absolute concentrations of polar metabolites in human plasma, which can be used in future as reference ranges in a Japanese population.

**Methods:**

Metabolomic profiling of 8,413 fasting plasma samples were completed using CE-MS, and 94 polar metabolites were structurally identified and quantified. Quality control (QC) samples were injected every ten samples and assessed throughout the analysis. Inter- and intra-batch coefficients of variation of QC and participant samples, and technical intraclass correlation coefficients were estimated. Passing-Bablok regression of plasma concentrations by CE-MS on serum concentrations by standard clinical chemistry assays was conducted for creatinine and uric acid.

**Results and conclusions:**

In QC samples, coefficient of variation was less than 20% for 64 metabolites, and less than 30% for 80 metabolites out of the 94 metabolites. Inter-batch coefficient of variation was less than 20% for 81 metabolites. Estimated technical intraclass correlation coefficient was above 0.75 for 67 metabolites. The slope of Passing-Bablok regression was estimated as 0.97 (95% confidence interval: 0.95, 0.98) for creatinine and 0.95 (0.92, 0.96) for uric acid. Compared to published data from other large cohort measurement platforms, reproducibility of metabolites common to the platforms was similar to or better than in the other studies. These results show that our CE-MS platform is suitable for conducting large-scale epidemiological studies.

## Introduction

Large-scale metabolomics in prospective epidemiological studies is a promising approach to identify biomarkers for prevention, diagnosis, and prognosis of chronic diseases including cardiovascular diseases [1–3] and cancer [4,5]. Since the metabolomic profile is indicative of biological alterations associated with a wide range of possible genetic or environmental factors, this is expected to give new insights to understand complex etiology of diseases, related to genes, external and internal environment, and their interactions [6,7].

Metabolomic profiling using liquid chromatography–mass spectrometry (LC-MS) and gas chromatography–mass spectrometry (GC-MS) has been conducted for over 1,000 blood samples collected in European cohorts including Cooperative Health Research in the Region of Augsburg (KORA) [6,8,9] and TwinsUK registry [6,8–10], and American cohorts such as Framingham Heart Study (FHS) Offspring cohort [2,3] and Atherosclerosis Risk in Communities Study (ARIC) [11]. Nuclear magnetic resonance (NMR) has also been used in population studies such as Estonian Biobank [12], Finnish cohorts [12], COMBI-BIO [13] and the INTERnational study of MAcro/micronutrients and blood Pressure (INTERMAP) Study [14–16]. However, large-scale cohorts involving 10,000s of individuals with metabolomics data are still limited. In addition, Asian cohorts with metabolomics are limited in scope or small-scale in size. It is essential to conduct large-scale metabolomics studies in different populations, as it has been reported that metabolomic profiles vary by ethnic group and lifestyle [15,17,18]. We therefore initiated the Tsuruoka Metabolomics Cohort Study (TMCS) [19–21] in Japan enrolling 11,002 participants since April 2012. This is among the first Japanese population-based cohort studies with metabolomics, using capillary electrophoresis-mass spectrometry (CE-MS) for polar metabolites and LC-MS for lipid metabolites.

Compared to other methods of metabolomic profiling, the CE-MS method has high separation efficiency and compound identification capability, and allows the absolute quantification of polar metabolites, including carbohydrates and amino acids [22–24]. Also, CE-MS has unique advantages which are suitable for large-scale epidemiological studies. Firstly, the ability for multiplex separations with serial injections of seven or more samples in a single run allows of higher sample throughput at lower costs with high quality assurance since a QC is included in every run [25]. Secondly, CE-MS is optimal to analyse volume-restricted biospecimens which is critical for retrospective analysis [26].

Some epidemiological or long-term studies using CE-MS were recently reported [27–28], however, reliability in measurement of thousands of blood samples over long periods of time is still unclear, especially inter-batch variations. In order to estimate a precise disease risk with high statistical power in epidemiological studies, it is critically important to limit measurement error and bias [29]. Thus, in this study, we aimed to examine the reproducibility and validity of large-scale CE-MS measurements and identify the compounds with reliable measurements.

It is also important to establish the absolute concentrations of metabolomics biomarkers in epidemiological studies because this helps us to compare and combine the results among different studies and to determine the levels to use practically for prevention. However, little population-based information has been available even for values according to sex and age [30]. Our CE-MS platform can yield these values for a wide range of polar metabolites.

In this study, we examined the reliability of large-scale metabolomics profiling via our CE-MS platform, using 883 quality control (QC) samples for cation metabolites and 946 for anions as well as 8,413 participant plasma samples, with data acquired over a period of 52 months. The measurement values for creatinine and uric acid were validated by comparison with independent clinical laboratory assays of these variables. In addition, we present summary data of absolute plasma concentration for each metabolite according to sex and age for community-dwelling adults in Japan, which may be used in future as reference values.

## Materials and methods

### Study population and sample collection

TMCS is a Japanese cohort, initiated in April 2012 (Tsuruoka City, Yamagata Prefecture, Japan), involving 11,002 participants aged 35 to 74 years. They were recruited among attendees of annual municipal or workplace health check-up programs held in four sites of the city at baseline (2012 April—2015 March). All participants gave written informed consent for this study; its protocol was approved by the Medical Ethics Committee of the School of Medicine, Keio University, Tokyo, Japan (approval no. 20110264).

All participants completed a comprehensive questionnaire on lifestyle, dietary habit and medical history. In addition, biological samples including serum, plasma, urine and DNA, and medical examination data by health check-up programs were collected at recruitment. These data and samples continue to be collected prospectively, when the participants undergo annual municipal or workplace health check-up programs. The follow-up survey to collect information for death, change of address and medical information including incidence of cardiovascular diseases and cancer is also conducted every year, using national records and hospital records.

TMCS is particularly designed to discover metabolomics biomarkers for common diseases and disorders, related to environmental and genetic factors. In order to have the optimal samples for our CE-MS metabolomics platform, we followed suitable protocols reported previously [19,20,31]. In brief, blood samples were collected in the morning after 12h-overnight fasting. Plasma samples were collected with ethylenediaminetetraacetic acid-2Na (EDTA-2Na) as an anticoagulant and kept at 4°C immediately after collection. The samples were centrifuged for 15 minutes (1,500g at 4°C) within 3 hours of collection, divided into aliquots, and preserved at 4°C until extraction of metabolites. Metabolite extraction from plasma was finished within 6 hours after collection to reduce the metabolic reactions in plasma, then the extract was stored at -80°C. Fifty μL of plasma was used for sample extraction. The extraction method has been detailed previously [32].

### Metabolomics measurement and quality control samples

Metabolomic profiling was conducted for fasting plasma samples via capillary electrophoresis time-of-flight mass spectrometry (CE-TOFMS). CE-TOFMS analysis of cationic and anionic metabolites was performed as described previously [23,33,34]. Raw data were processed using our proprietary software (MasterHands) [34,35]. We used two CE-MS instruments to measure cation and two for anions exclusively. These four instruments were solely used for this study during the study period. Mass calibration using tuning solution and MS entrance cleaning were performed at the beginning of every sequence to ensure robust performance. In addition, in order to avoid unexpected changes in sensitivity or variance in measurement of mass in a continuous run, the number of samples per one run was limited up to 100. The average number of sample runs was 30 per day, and about 0.5% of runs failed due to current drop or capillary fracture in this study. As a preliminary study, we identified 154 polar metabolites with standard compounds in plasma. For all the participant samples, we measured absolute concentrations of 94 metabolites (54 cations and 40 anions, listed in S1 Table), which were expected to be detected in more than 20% of plasma samples.

Metabolomic profiles of participant samples were analysed from June 2012 in collected order, and completed for 8,413 samples until August 2016. These data consisted of 105 running batches of cations and 99 batches of anions. One batch contains an average of 80.1 samples (maximum 164) for cations, and 83.2 samples (maximum 168) for anions. To monitor the stability of metabolomics analysis, QC samples were injected every 10 samples and assessed at the start of the analytical run and at intervals throughout the analysis. In total, 883 QC samples for cations and 946 for anions were used for this study. For QC samples, 150 mL serum collected from 20 people from the same population in advance were extracted for metabolomics analysis as soon as collected, then divided into 50 μL aliquots and stored at -80°C. QC aliquots stored at -80°C were thawed and used for monitoring daily during the study. We calculated the mean concentration for each metabolite in QC samples which were previously analysed in 70 sequences, then when the concentration of each metabolite in QC samples continuously exceeded (more than twice) the mean concentration ± two standard deviations for more than half metabolites, we re-analysed the subsequent samples of sequence.

### Clinical laboratory assay

For the purpose of validating the absolute measurement values, we used standard clinical laboratory assay data for serum creatinine and uric acid. These data were collected from the health check-up programs that 2,325 of participants underwent in Tsuruoka Kyoritsu Hospital, at the same time as recruitment and metabolomics sample collection. Creatinine was measured by enzymatic method which is widely used in medical examinations in Japan [36] [37]. Uric acid was also measured by enzymatic method [38]. Both of these methods were authorised by the Japan Society of Clinical Chemistry as the national standardized method. Tsuruoka Kyoritsu Hospital acquired certification in quality control of these methods by the Japanese Association of Medical Technologists [39].

### Statistical analyses

For samples where metabolites were not detected, half of the lowest detected values were imputed [40]. Inter- and intra-batch variance for each metabolite concentration of QC samples were computed to evaluate reproducibility, using a linear mixed model with observed metabolite level, Y, and a random effects common to each batch, B.

$$Y_{i}=\mu +B_{i}+\varepsilon_{i}$$

Then, we calculated the coefficient of variation (CV), by dividing the variance estimated from this model by the mean. Pearson correlation coefficients between inter- and intra-batch CV were also calculated. These analyses were also conducted with participant samples to assess inter- and intra- batch variance.

The intraclass correlation coefficient (ICC) was calculated to compare the reliability of the metabolomics biomarkers with previous research [41,42]; it was calculated from variance of measurement errors, σ_E_^2^, and total variance, σ_T_^2^.

$$ICC=1-\frac{\sigma {\;}_{E}^{2}}{\sigma {\;}_{T}^{2}}$$

Although we could not compute ICC for participant samples as there were no replicates, we computed technical errors from the large number of replicates for QC samples considered to be representative of the population samples. We made approximate calculation of ICC, substituting CV of QC samples for error variance and CV of participant samples for total variance.

$$Approximate\;ICC=1-\frac{{\left({CV}_{QC}\right)}^{2}}{{\left({CV}_{Participant}\right)}^{2}}$$

We conducted Passing-Bablok regression of our CE-MS measurements in plasma on standard clinical laboratory measurements in serum for creatinine and uric acid concentrations. We also showed Bland-Altman plots using mean of these two methods and percentage of differences.

We summarized the metabolomics data stratified by sex and age. Linear regression analysis was performed to investigate differences by sex and age, with adjustment for possible confounders: smoking and alcohol drinking habit, history of any ischemic heart disease, stroke and cancer, and current disease status including hypertension, diabetes, dyslipidaemia and impaired kidney function. Bonferroni correction was used to account for multiple testing (α = 0.05/94).

All statistical analyses were performed using performed using R.3.3.1 (R Core Team 2016, R Foundation for Statistical Computing, Vienna, Austria.).

## Results

### Coefficient of variation for quality control samples

**Fig 1:**
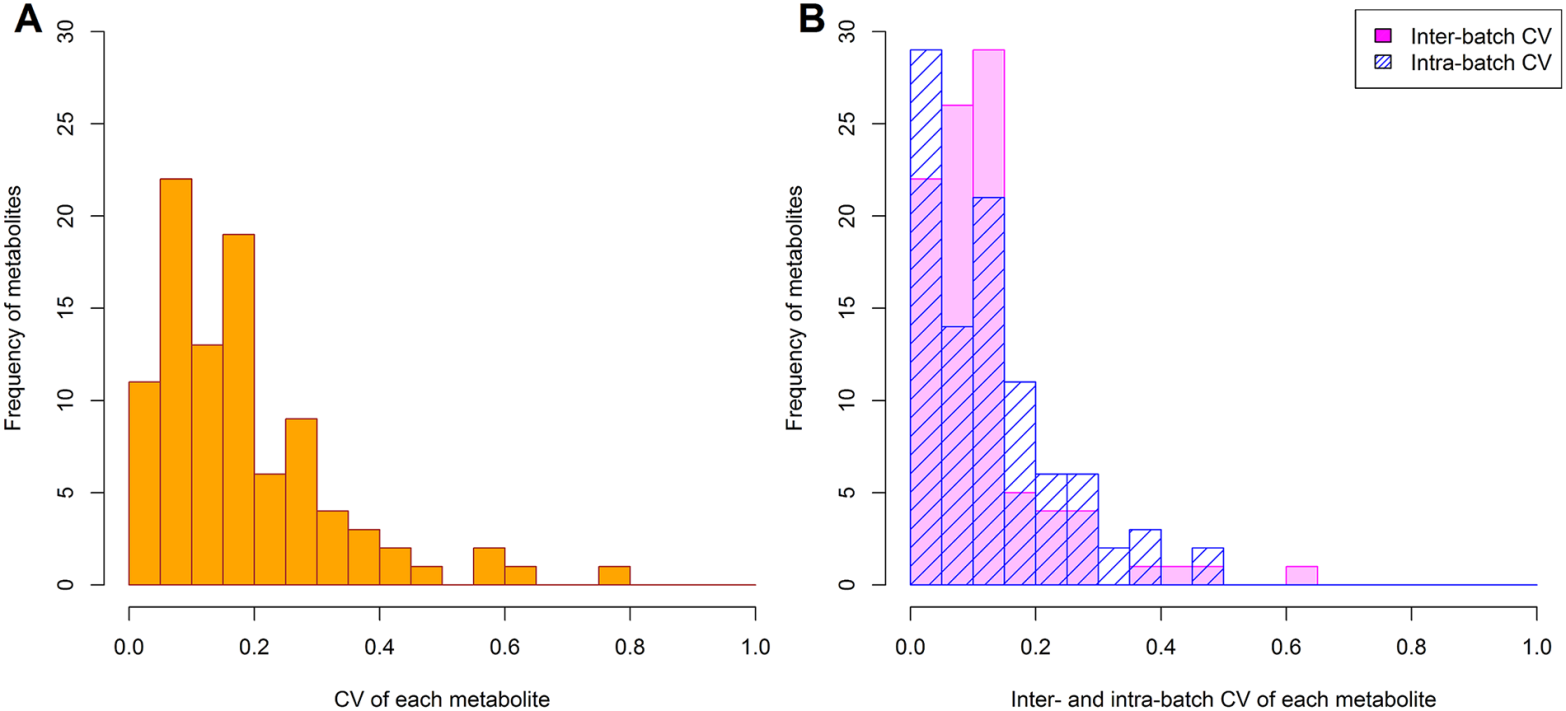
Histogram of CV for 94 metabolites in QC samples. (A) Coefficients of variation (CV) for detected 94 metabolites in quality control (QC) samples. (B) Inter- and intra-batch CV for each metabolite in QC samples. Inter- and intra-batch CV were computed using linear mixed models.

CV values for QC samples are shown in S1 Table. Of the 94 metabolites, CV was less than 20% for 64 metabolites (68%), 20–30% for 16 metabolites (17%), and more than 30% for 14 (16%) (Fig 1A). Median CV was 7.9% for cation compounds and 18.9% for anions. Boxplots of metabolite concentrations by batches were shown in S1 and S2 Files.

The comparison of reproducibility with other major MS-platforms used in large epidemiologic studies was shown in Table 1 and S2 Table. CV values of overlapping polar metabolites were similar to or better than in other platforms.

**Table 1:** Comparison of reproducibility for polar metabolites between major MS platforms used in large cohorts.

	Platform 1	Platform 2	Platform 3	Platform 4
Laboratory performing analysis	IAB, Keio University	Metabolon Inc.	GAC, Helmholtz Zentrum München	Broad Institute
Study showing CV in QC samples for polar metabolites	This study	Shin *et al*., 2014 [9]	Illig *et al*., 2010 [43]	Shaham *et al*., 2008 [44]
Cohorts of the above study	Tsuruoka Metabolomics Cohort	KORA and Twins UK	KORA and Twins UK	FHS Offspring cohort
Separation method for polar metabolites	CE	LC and GC	LC using Absolute IDQ^™^ kit (BIOCRATES Life Sciences AG)	LC
N overlapping metabolites with Platform 1	94 (reference)	58	15	40
Median of CV for metabolites in Platform 1	15.5%	-	-	-
for overlapping with Platform 2	10.5%	9.6%	-	-
for overlapping with Platform 3	6.0%	-	6.9%	-
for overlapping with Platform 4	10.7%	-	-	16%
How to measure QC samples	QC samples were injected every 10 subject samples, corresponding to 1829 QC samples for the study.	4–5 replicates were run each platform day, corresponding to 1300 QC samples for the study.	45 technical replicates were measured on 9 different kit plates (5 replicate samples per kit).	CV was assessed by splitting one plasma sample into 7 parts and processing each part separately.

CE: capillary electrophoresis, CV: coefficient of variation, FHS: Framingham Heart Study, GAC: Genome Analysis Centre, GC: Gas chromatography, IAB: Institute for Advanced Biosciences, KORA: Cooperative Health Research in the Region of Augsburg, LC: liquid chromatography, MS: mass spectrometry, QC: quality control.

Inter-batch CV estimated via a linear mixed model was less than 20% for 81 compounds (86%), but more than 30% for four of them. Intra-batch CV was less than 20% for 74 compounds (78%) (Fig 1B). Inter- and intra-batch CV had similar values (medians, respectively 5.8% and 5.0% for cations; 11.9% and 13.8% for anions). They were also highly correlated (Pearson’s r = 0.85) (S1 Fig).

### Variation for participant samples

**Fig 2:**
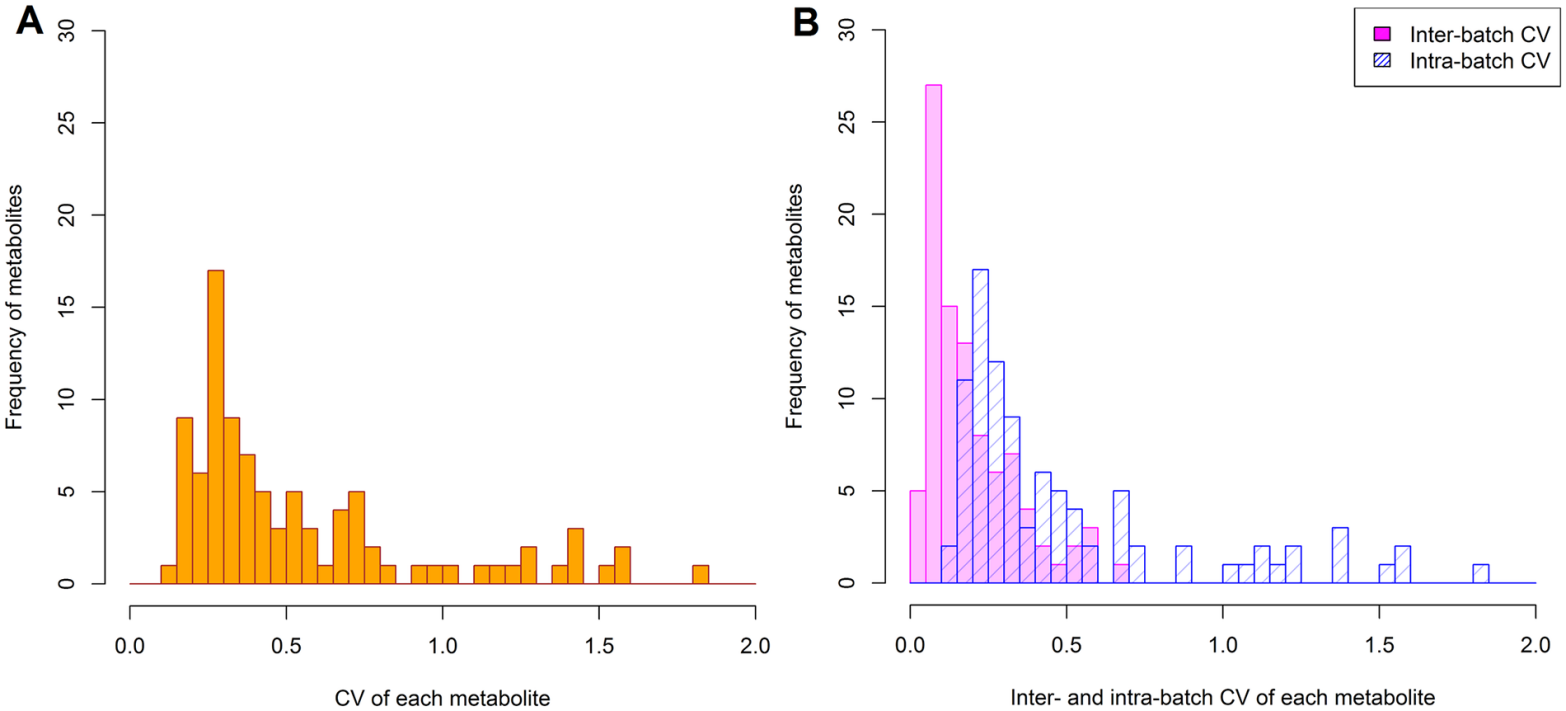
Histogram of CV for each metabolite in participant samples. (A) Coefficient of variation (CV) for each detected metabolite in participant plasma samples. (B) Inter- and intra-batch CV for each metabolite in participant samples. Inter- and intra-batch CV were computed using linear mixed models.

Statistical summary of metabolites measured in participant samples is shown in S1 Table. Total, intra- and inter- batch CV among participants are shown in Fig 2. Medians of total, inter- and intra-batch CV were 32.3%, 9.9% and 30.4% for cation metabolites, respectively, and 44.9%, 20.2%, and 38.4% for anions. As expected, CV in participant samples was larger than in QCs. Participant samples had larger intra-batch CV than inter-batch CV, in contrast to QCs.

**Fig 3:**
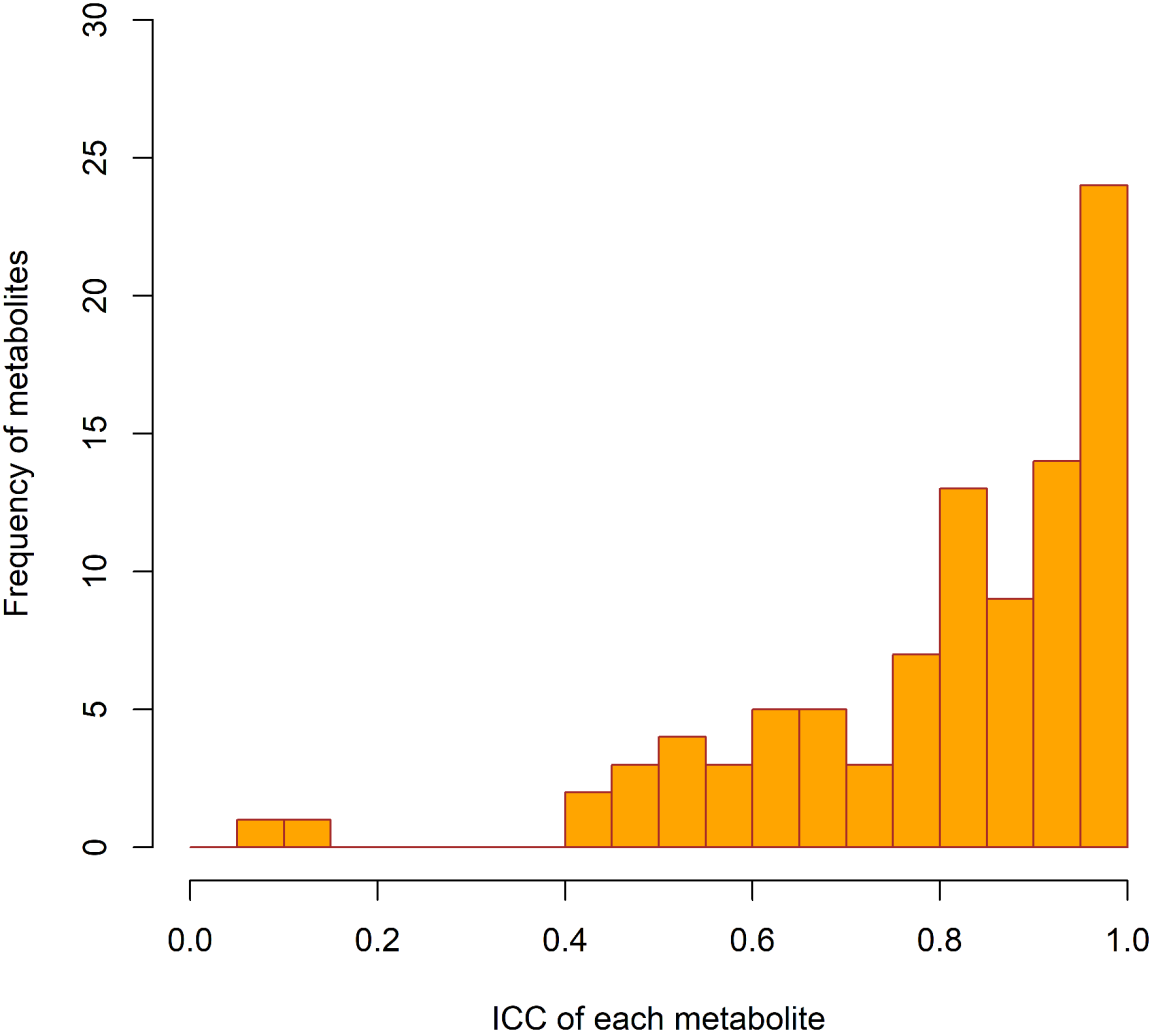
Histogram of estimated ICC. Estimated intraclass correlation coefficients (ICC) calculated using the formula, 1 − (Total CV of QC samples)^2^ / (Total CV of subject samples)^2^.

Results of calculation of estimated ICC are shown in Fig 3 and S1 Table. This was > 0.75 for 67 metabolites (71%), 0.40–0.75 for 25 metabolites (27%), and < 0.40 for two (uridine and malonate, 2%).

**Fig 4:**
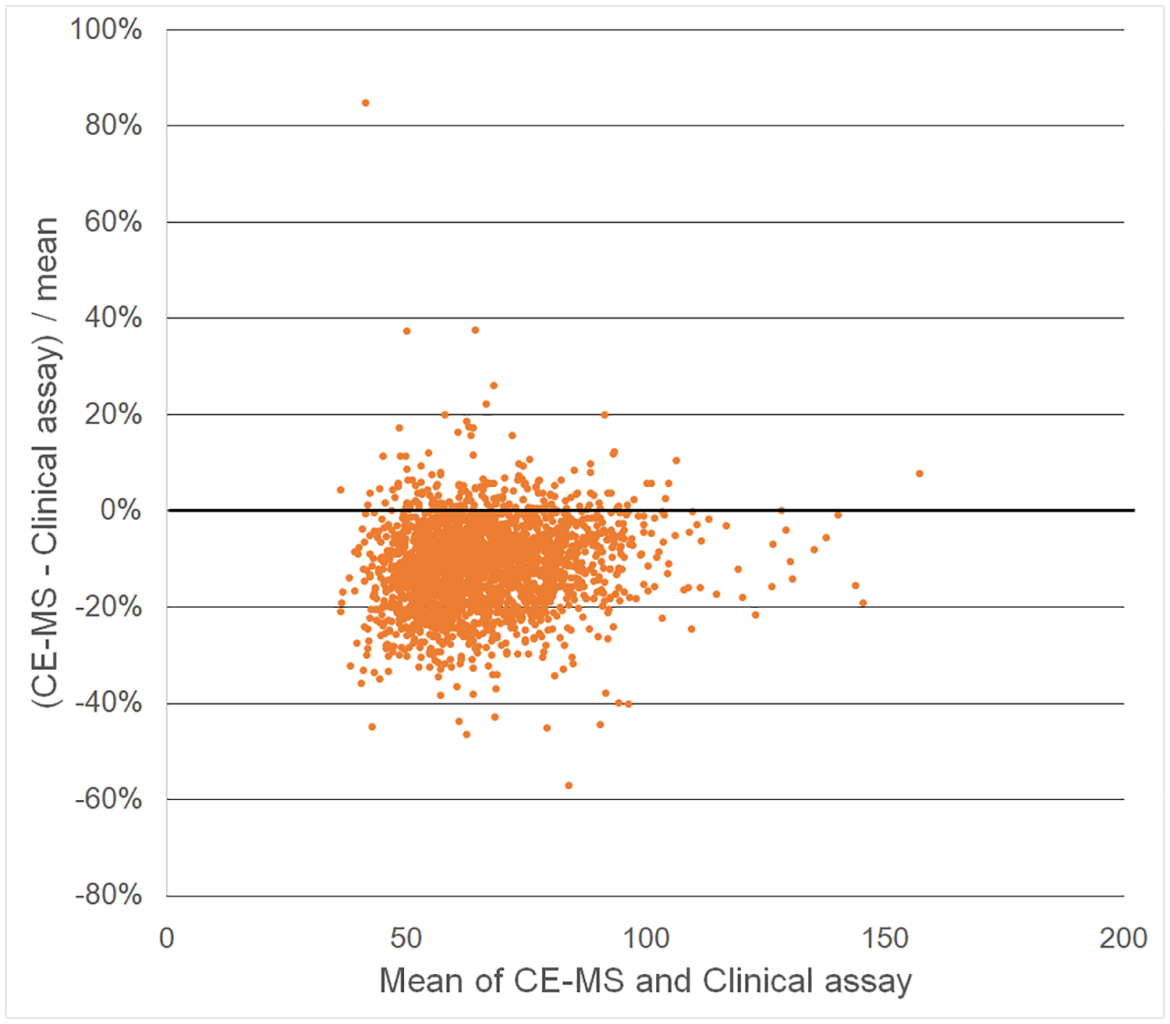
Bland-Altman plots for creatinine. X-axis indicates the mean creatinine concentrations (μmol/L) of capillary electrophoresis-mass spectrometry (CE-MS) and clinical assay, and Y-axis indicates percentage of differences between these two methods.

**Fig 5:**
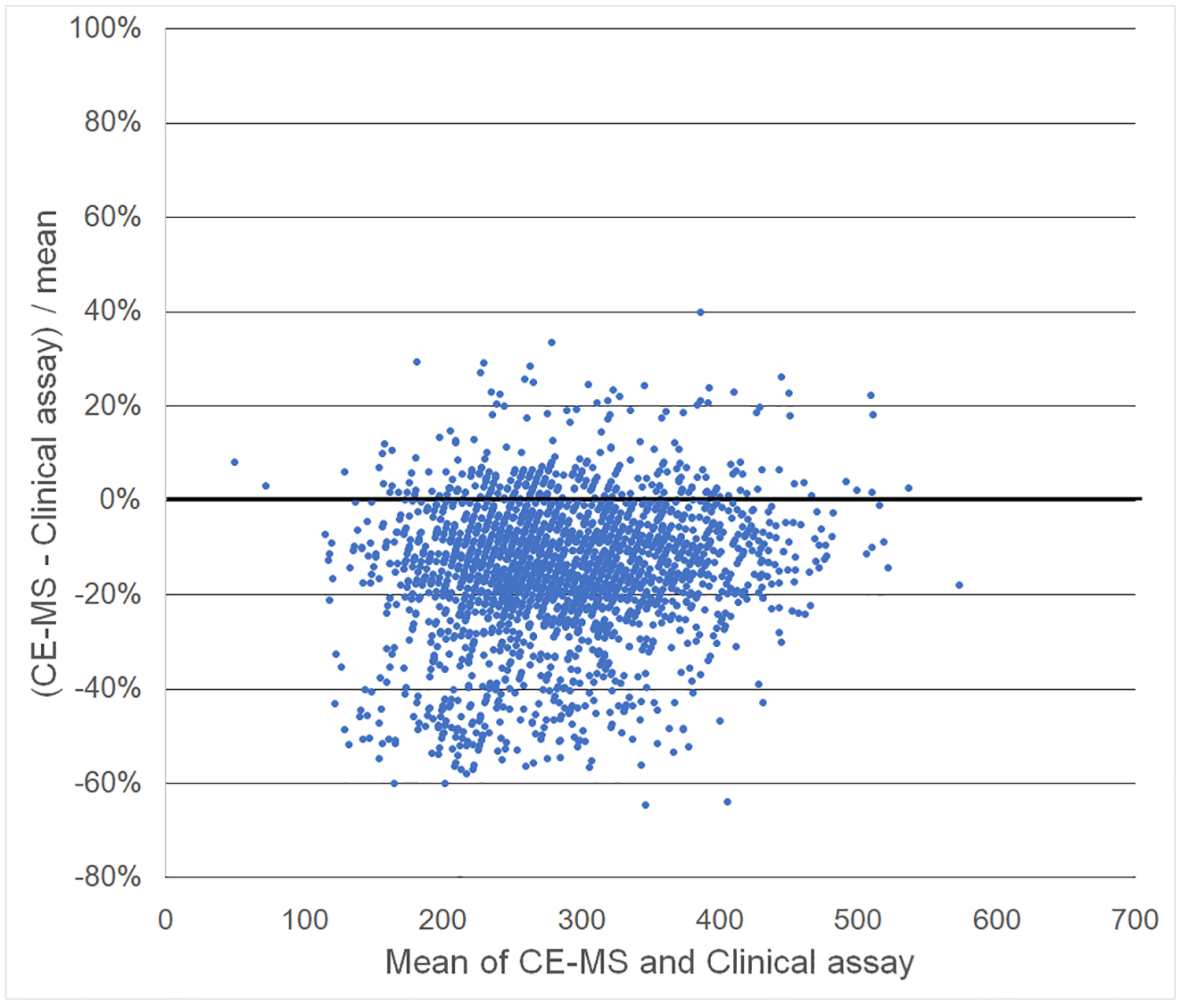
Bland-Altman plots for uric acid. X-axis indicates the mean uric acid concentrations (μmol/L) of capillary electrophoresis-mass spectrometry (CE-MS) and clinical assay, and Y-axis indicates percentage of differences between these two methods.

Passing-Bablok regression of CE-MS measurements on standard clinical laboratory measurements estimated slope 0.97 (95% confidence interval: 0.95, 0.98) and intercept -4.52 (-5.69, -3.37) for creatinine, and slope 0.95 (0.92, 0.96) and intercept -21.03 (-27.15, -15.36) for uric acid. This result for creatinine was consistent even after excluding three samples more than five standard deviations from the mean as outliers (slope = 0.97, intercept = -21.19). As shown in intercept values, absolute concentrations of these were relatively lower for plasma by CE-MS than for serum by the independent clinical assay (Creatinine mean ± standard deviation: 63.2±20.3 μmol/L by CE-MS vs 70.4±22.0 μmol/L by clinical assay, p < 0.001 for paired t-test; Uric acid: 266.5±72.9 μmol/L vs 307.6±75.6 μmol/L, p < 0.001). Bland-Altman plots also shows that CE-MS provided lower values than clinical assay, whereas any other bias was unlikely to be observed. (Figs 4 and 5).

Table 2 and S3 Table show results by sex and age. 73 metabolites differed by sex after age adjustment and Bonferroni correction. 49 compounds for males and 64 for females were significantly related to age. Analysis of creatinine and uric acid measured by clinical assay showed similar results to CE-MS (Table 2).

**Table 2:** Concentrations of creatinine and uric acid stratified with sex and age.

	All ages	Difference by sex		-44 y	45–54 y	55–64 y	65- y	Difference per age	
Male	Mean (μmol/L)	Estimate	P value	Mean (μmol/L)	Mean (μmol/L)	Mean (μmol/L)	Mean (μmol/L)	Estimate	P value
N	1102			28	93	397	584		
Creatinine (CE-MS)	72.64±24.43	17.89	1.75E-110	73.74±10.04	70.34±13.54	71.26±13.20	73.90±31.16	0.11	2.80E-01
Creatinine (Clinical assay)	80.38±26.64	18.95	3.64E-105	80.79±8.75	77.46±14.33	78.53±13.44	82.08±34.28	0.17	1.19E-01
Uric acid (CE-MS)	298.53±71.96	60.74	1.70E-98	307.84±64.75	317.13±76.81	306.06±74.16	290.00±68.89	-1.28	2.20E-05
Uric acid (Clinical assay)	345.87±71.34	72.54	5.04E-134	355.18±58.23	364.04±75.19	350.36±70.96	339.47±70.92	-1.21	5.50E-05
**Female**									
N	1223			17	104	463	639		
Creatinine (CE-MS)	54.77±9.82	ref	ref	57.45±7.48	53.65±9.41	54.42±9.70	55.13±10.02	0.05	2.23E-01
Creatinine (Clinical assay)	61.46±10.63	ref	ref	63.08±8.83	60.35±9.52	60.92±10.35	61.99±11.03	0.08	6.73E-02
Uric acid (CE-MS)	237.62±60.74	ref	ref	242.26±65.28	240.63±62.53	235.67±58.45	238.42±62.03	-0.11	6.59E-01
Uric acid (Clinical assay)	273.2±61.58	ref	ref	268.36±70.87	273.61±56.84	271.66±59.60	274.39±63.57	0.12	6.37E-01

CE-MS: capillary electrophoresis-mass spectrometry, ref: reference

## Discussion

In our large-scale epidemiological study using the CE-MS metabolomics platform we report concentrations for 94 polar compounds in blood with good to high reproducibility: CV for 80 compounds (85% of all) was less than 30% despite a measurement period of 52 months. Inter-batch CV was less than 20% for 81 compounds (86%) among 105 batches for cations and 99 batches for anions. The measured values by our CE-MS method for creatinine and uric acid were similar to established clinical laboratory assays for these compounds widely used in epidemiologic studies.

In metabolomics studies, QC sample methods are widely used to evaluate reproducibility [45]. Features with QC CV < 20% are often considered to have good reproducibility, as recommended by the US FDA [46]. Features with QC CV < 30% are also considered acceptable [45,47]. Compounds with lower reproducibility in our analyses had small peaks and low signal/noise ratios, therefore, it was difficult to detect these peak areas precisely, and to differentiate them from noise.

Metabolon [9,10] and the Broad institute [2,44,48] have conducted large-scale targeted metabolomics measurement for cohort studies including KORA, Twins UK, ARIC and FHS Offspring Cohort. Absolute IDQ^™^ kits (BIOCRATES Life Sciences, Innsbruck, Austria) have been used by studies like KORA and Twins UK [43]. Compared to published data from these platforms, reproducibility in this study which has unique broad coverage of polar metabolites was similar to or better than in other studies.

In order to reduce measurement errors, we strictly limited the instruments used for this study, and were checking sensitivity of instruments regularly. Also, we reanalyse samples when monitored QC sample concentrations did not match the criteria, in order to keep measurement quality. These careful settings might contribute to good reliability equally to other metabolomics platforms.

However, this comparison should be treated with caution as the method for calculating CV was different between platforms especially for measurement duration and number of batches and replicates. Nonetheless, this result shows that our platform is at least comparable to others and suitable for conducting large-scale epidemiological studies. It should be noted that our values are reported as absolute concentrations of compounds, instead of the relative intensity of features.

Compared to other established MS platforms, our CE-MS platform has limited coverage for overall metabolites because CE-MS is not able to detect most of non-polar metabolites. Most of polar metabolites were detected consistently in participants, however, some metabolites (especially anions) were hard to be quantified with adequate precision due to their lower concentrations than limit of detection. Despite this limitation, our platform still has broader coverage of polar metabolites than other MS platforms. In addition, environmental load and cost is relatively lower because little organic solvent is used in CE-MS. Compared to NMR, CE-MS has lower sample throughput, but coverage and sensitivity for polar metabolites are higher. Therefore, CE-MS is also suitable to use in combination with other methods to cover broader metabolites.

Variations between samples can be classified as inter- or intra-batch variations. Reducing inter-batch variations is an important issue in large-scale metabolomics [49,50]. The results show that our metabolomics method controlled inter-batch effects well for most of the measured compounds without any statistical adjustments, regardless of the large scale with 883 QC samples among 105 batches for cations and 946 QC samples among 99 batches for anions. Inter- and intra-batch variations were comparable. Even for metabolites with poor reproducibility, this reflected intra-batch effects, except for triethanolamine, indole-3-acetate, and malonate where inter-batch CV was larger than intra-batch one.

The larger CV for participant plasma samples than for serum QCs can be interpreted as due to variation between subjects including biological differences. The larger intra-batch CV than inter-batch CV also indicates that increasing CV for participant samples was mainly due to the variation between subjects, rather than measurement errors. Although the difference between serum and plasma should be taken into account, our preliminary study showed that the metabolites detected in serum samples included all the metabolites detected in plasma, and metabolite concentrations in EDTA plasma and serum correlate well with the exception of some metabolites such as hypoxanthine and lactic acid [31,51].

Technical ICC can add another aspect to evaluate technical variation, focusing on participant variation including true biological differences. In epidemiological studies, statistical power is influenced by the magnitude of the effect of interest in the population. When the biological variation is larger than technical variation, biological differences can be detected despite measurement errors. Our estimate of ICC, 1 − (Total CV of QC samples)^2^ / (Total CV of Participant samples)^2^, was above 0.40 for most measured compounds, except for malonate and uridine. Even for compounds with poor reproducibility, those with ICC above 0.40 may be worth examining as potential biomarkers, with careful evaluation of their measurement errors.

The reliability of CE-MS measurements for creatinine and uric acid, measured by cation mode and anion mode, respectively, were evaluated with respect to clinical laboratory data. Clinical measurements of serum creatinine and uric acid are standardized and certificated nationwide, and commonly adopted in medical laboratories and hospitals. The slope of Passing Bablok regression was nearly one with narrow 95% confidence interval in both of creatinine and uric acid. These results indicate that CE-MS and clinical laboratory data were similar, although the slope was slightly less than one and the intercept was less than zero due to the 10–13% lower mean concentrations by CE-MS. It is possible that this was due to the difference between plasma and serum, considering previously reported findings that most metabolite concentrations are higher in serum than in EDTA plasma [51]. Nonetheless both methods gave similar results with respect to differences by sex and age. This further indicates that our metabolomics data are fit for purpose for epidemiological studies.

Our metabolomics summary data by sex and age provide for the first time absolute concentrations for a large sample of community-dwelling adults in Japan. These data can be used as a reference for other population studies, especially Asians, though careful interpretation is required for some of the compounds with low reproducibility. Our data show sex differences in concentrations for most metabolites even after adjusting for potential confounders including unique metabolites in our CE-MS platform such as guanidinoacetate, gamma-butyrobetaine, and mucate that may contribute to explaining the biological sex differences. Many metabolites were also associated with age after adjustment for confounders, although we are unable to evaluate whether this is cause or consequence because of the cross-sectional design.

This study is characterized by large sample size from one population of community dwelling adults in Tsuruoka city, Japan. This can result in good internal validity in this population. This population can be considered as representative of Japanese whose genetic background is homogeneous, although the variety of environmental factors should be taken account of. In order to generalize our findings, further studies are needed in other populations in Japan and other countries. In addition, external validation and cross-platform and inter-lab round-robin studies are expected to establish the methodology of large-scale studies using CE-MS platform. International collaborative studies should be proceeded to address these issues.

In conclusion, this study shows the CE-MS platform yields reliable concentrations for plasma metabolites in a large-scale study. It provides high-quality metabolomics data which will aid in the understanding of links between disease risk and metabolism.

## Supporting information

S1 Fig Correlation between inter- and intra- batch CV in QC samples. The plots between inter- and intra- batch coefficients of variation in quality control (QC) samples are shown.
(TIFF)

S1 File Boxplots of cationic metabolite concentrations by batches in QC samples. (PDF)

S2 File Boxplots of anionic metabolite concentrations by batches in QC samples. (PDF)

S1 Table Statistical summary of measured metabolites. CV: coefficient of variation, ICC: intraclass correlation coefficient, ND: not detectable values, QC: quality control, SD: standard deviation. *ICC was estimated by following; 1 − (Total CV of QC samples)^2^ / (Total CV of subject samples)^2^.
(XLSX)

S2 Table Comparison of CV for polar metabolites in QC samples between major mass spectrometry metabolomics platforms used in large cohort studies. CE: capillary electrophoresis, CV: coefficient of variation, FHS: Framingham Heart Study, GAC: Genome Analysis Centre, GC: Gas chromatography, IAB: Institute for Advanced Biosciences, KORA: Cooperative Health Research in the Region of Augsburg, LC: liquid chromatography, QC: quality control. *1 CV of this detected metabolite was not reported. *2 CV of asymmetric dimethylarginine and symmetric dimethylarginine was not reported separately. *3 CV of leucine and isoleucine was not reported separately. *4 CV of 2-oxoglutarate and adipate was not reported separately. *5 CV of citrate and isocitrate was not reported separately. *6 CV of fumarate and malate was not reported separately.
(XLSX)

S3 Table Concentrations of all metabolites stratified with sex and age. SD: standard deviation, SE: standard error. *Confounders are following; smoking and alcohol drinking habit, history of any ischemic heart disease, stroke and cancer, and current disease status including hypertension, diabetes, dyslipidaemia and impaired kidney function. Hypertension was defined as systolic blood pressure ≥ 140 mmHg and/or diastolic blood pressure ≥ 90 mmHg and/or currently on antihypertensive therapy; impaired glucose tolerance as fasting plasma glucose ≥ 126mg mg/dL and/or hemoglobin A1c (NGSP) ≥ 6.5% and/or current use of antidiabetic medication.
(XLSX)
