# Supplementary figures and images for: Reliability of plasma polar metabolite concentrations in a large-scale cohort study using capillary electrophoresis-mass spectrometry
Source: PLoS One. 2018 Jan 18;13(1):e0191230. doi: 10.1371/journal.pone.0191230 (PMC5773198; doi:10.1371/journal.pone.0191230)

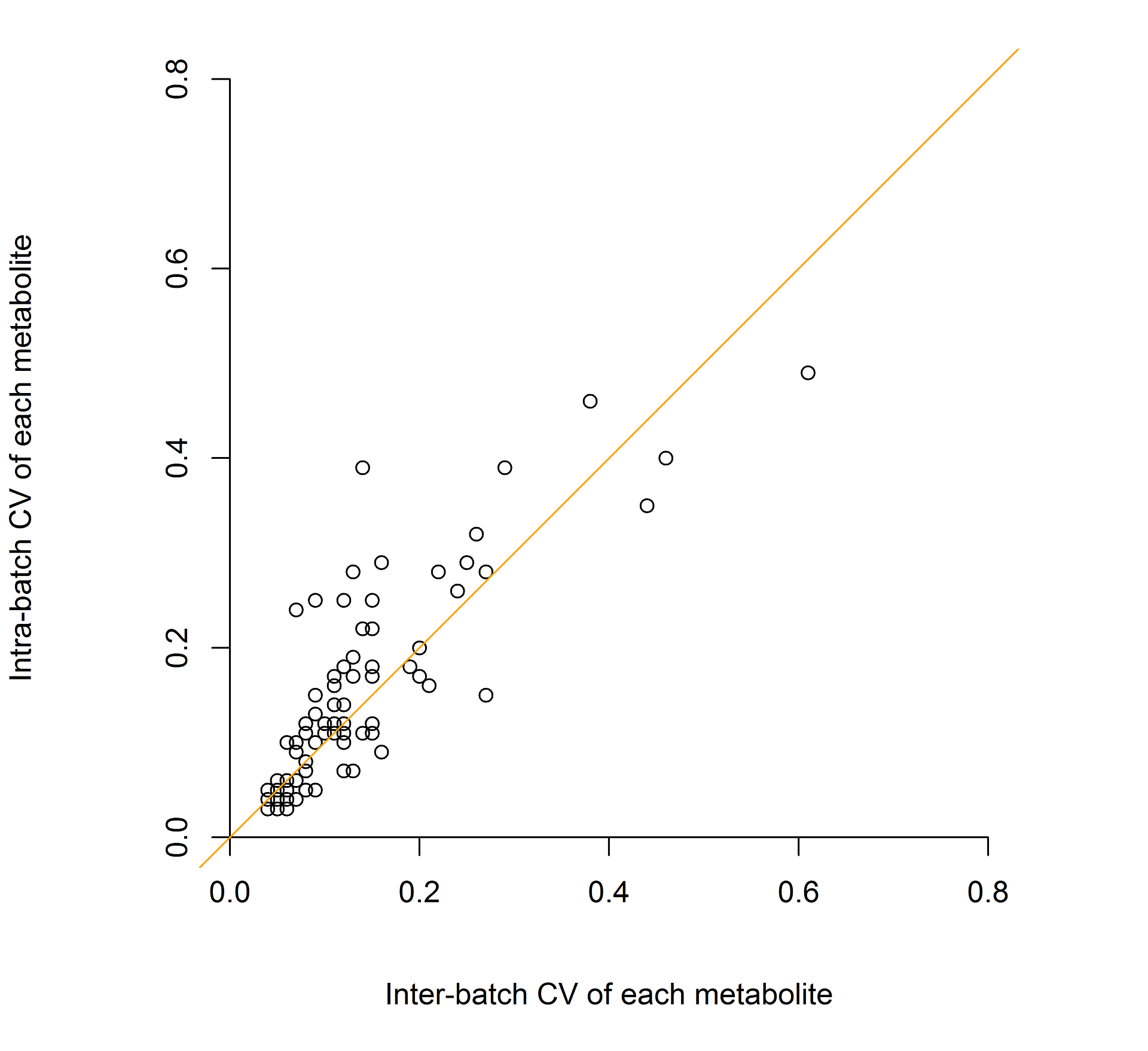

Supplement: S1 Fig — The plots between inter- and intra- batch coefficients of variation in quality control (QC) samples are shown. (TIFF) [file pone.0191230.s001.tiff]
