# Supplementary material for: Reliability of plasma polar metabolite concentrations in a large-scale cohort study using capillary electrophoresis-mass spectrometry
Source: PLoS One. 2018 Jan 18;13(1):e0191230. doi: 10.1371/journal.pone.0191230 (PMC5773198; doi:10.1371/journal.pone.0191230)
Supplement: S1 File — (PDF) [file pone.0191230.s002.pdf]

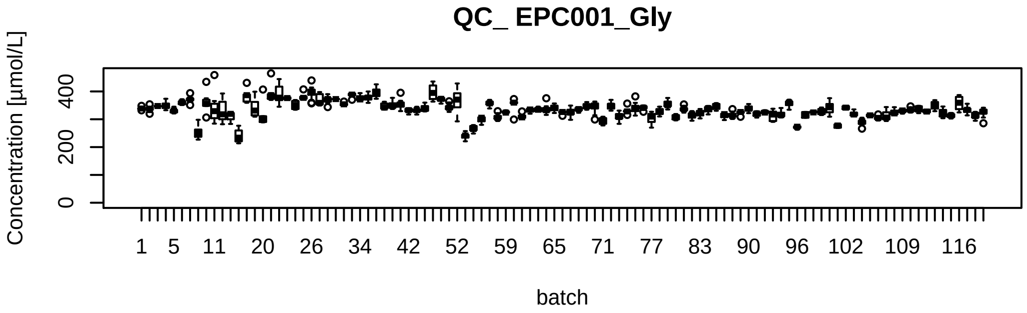
QC_ EPC001_Gly
Concentration [µmol/L]
0 200 400
1 5 11 20 26 34 42 52 59 65 71 77 83 90 96 102 109 116
batch

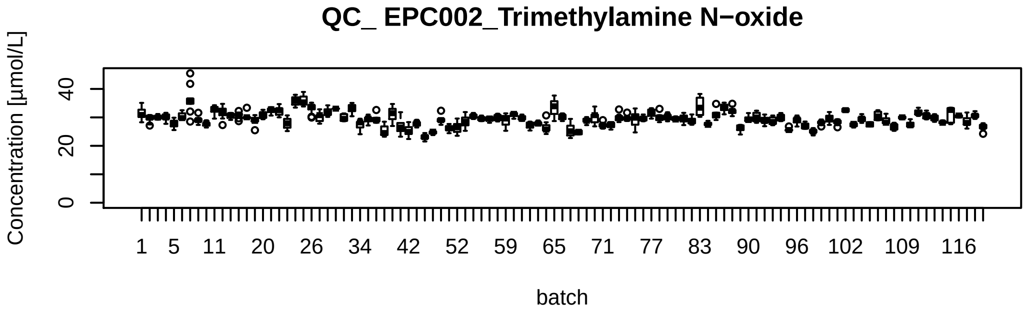
QC_ EPC002_Trimethylamine N−oxide
Concentration [µmol/L]
0 20 40
1 5 11 20 26 34 42 52 59 65 71 77 83 90 96 102 109 116
batch

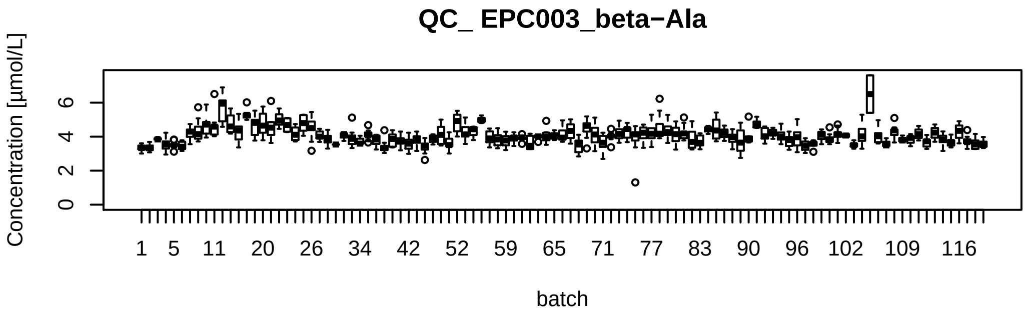
QC_ EPC003_beta−Ala
Concentration [µmol/L]
0 2 4 6
1 5 11 20 26 34 42 52 59 65 71 77 83 90 96 102 109 116
batch

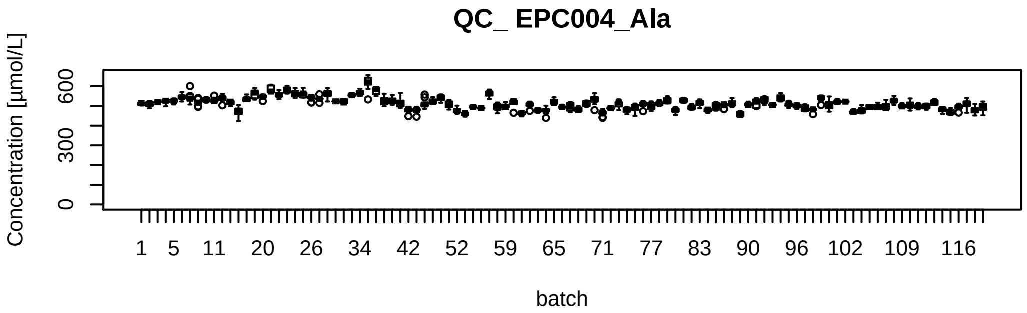
QC_ EPC004_Ala
Concentration [µmol/L]
0 300 600
1 5 11 20 26 34 42 52 59 65 71 77 83 90 96 102 109 116
batch

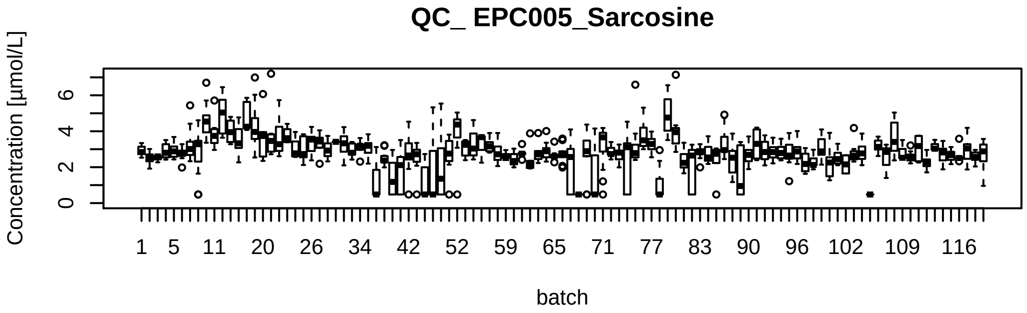
QC_ EPC005_Sarcosine
Concentration [µmol/L]
0 2 4 6
1 5 11 20 26 34 42 52 59 65 71 77 83 90 96 102 109 116
batch

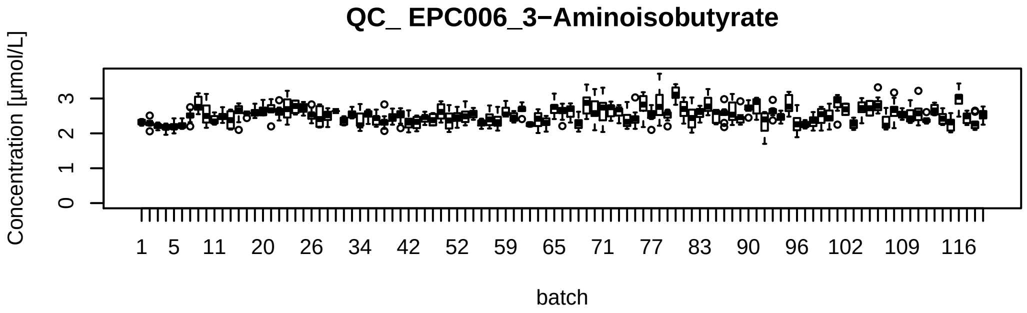
QC_ EPC006_3−Aminoisobutyrate
Concentration [µmol/L]
0 1 2 3
1 5 11 20 26 34 42 52 59 65 71 77 83 90 96 102 109 116
batch

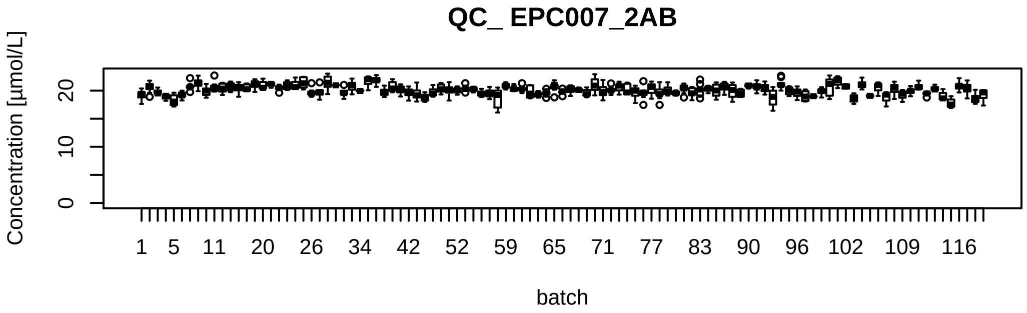
QC_ EPC007_2AB
Concentration [µmol/L]
0 10 20
1 5 11 20 26 34 42 52 59 65 71 77 83 90 96 102 109 116
batch

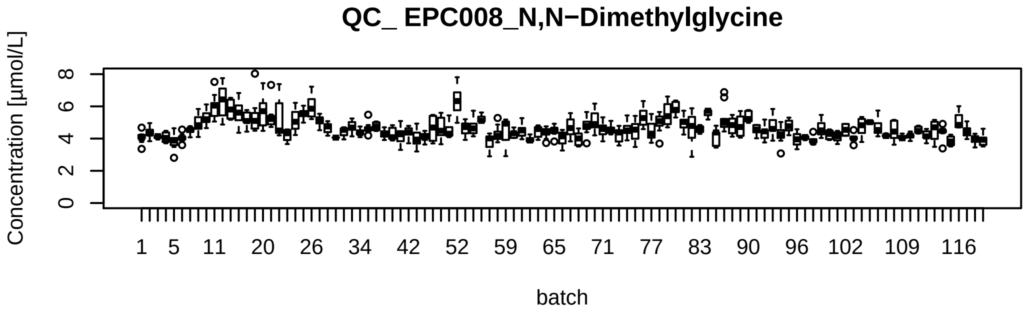
QC_ EPC008_N,N−Dimethylglycine
Concentration [µmol/L]
0 2 4 6 8
1 5 11 20 26 34 42 52 59 65 71 77 83 90 96 102 109 116
batch

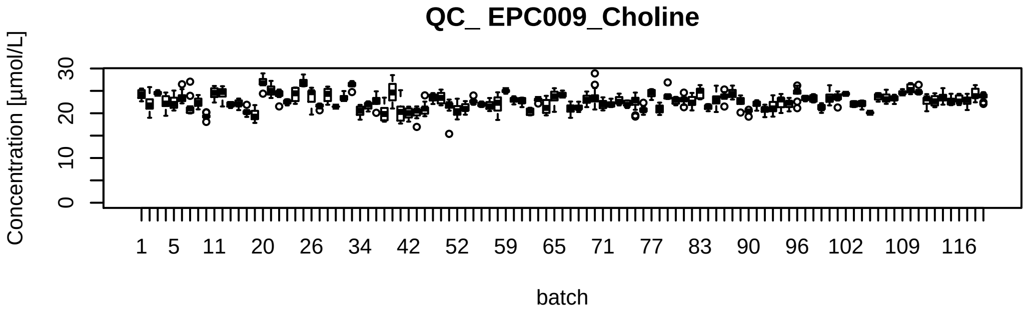
QC_ EPC009_Choline
Concentration [µmol/L]
0 10 20 30
1 5 11 20 26 34 42 52 59 65 71 77 83 90 96 102 109 116
batch

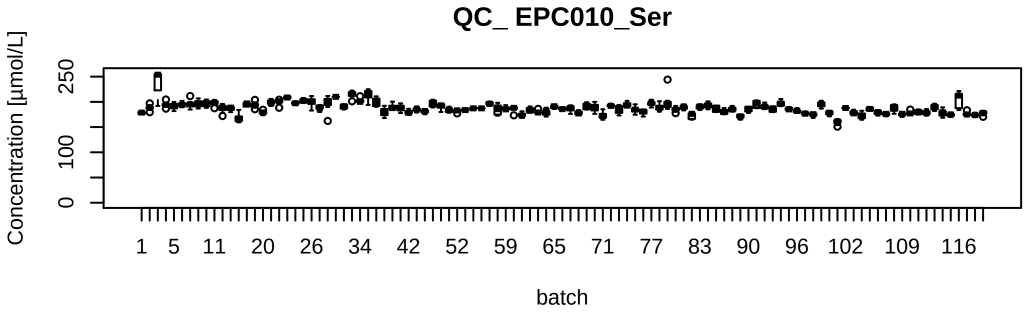
QC_ EPC010_Ser
Concentration [µmol/L]
0 100 250
1 5 11 20 26 34 42 52 59 65 71 77 83 90 96 102 109 116
batch

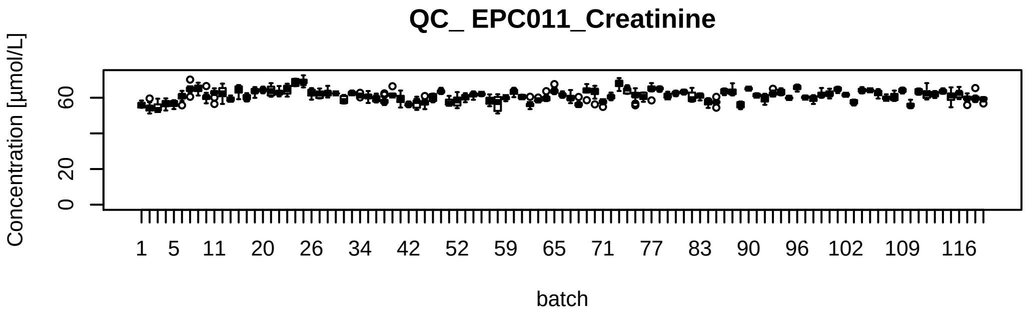
QC_ EPC011_Creatinine
Concentration [µmol/L]
0 20 60
1 5 11 20 26 34 42 52 59 65 71 77 83 90 96 102 109 116
batch

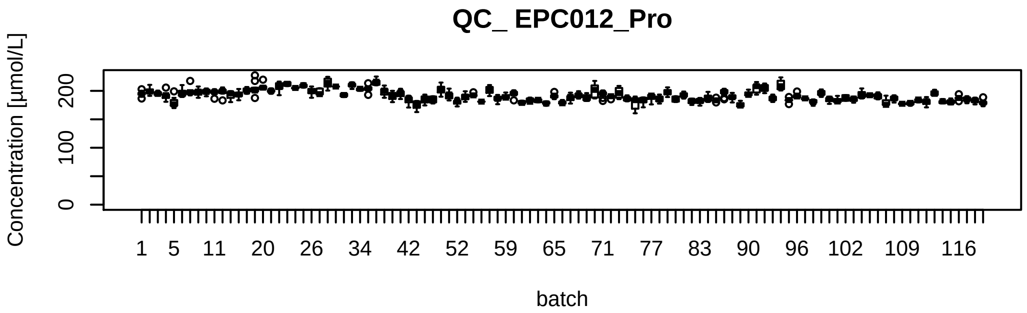
QC_ EPC012_Pro
Concentration [µmol/L]
0 100 200
1 5 11 20 26 34 42 52 59 65 71 77 83 90 96 102 109 116
batch

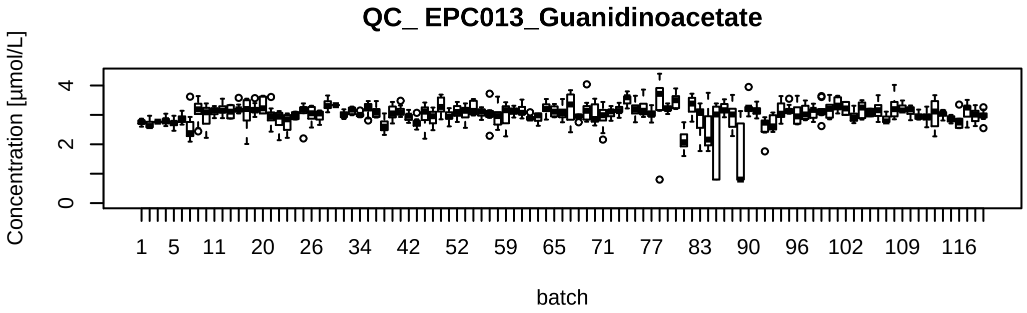
QC_ EPC013_Guanidinoacetate
Concentration [µmol/L]
0 2 4
1 5 11 20 26 34 42 52 59 65 71 77 83 90 96 102 109 116
batch

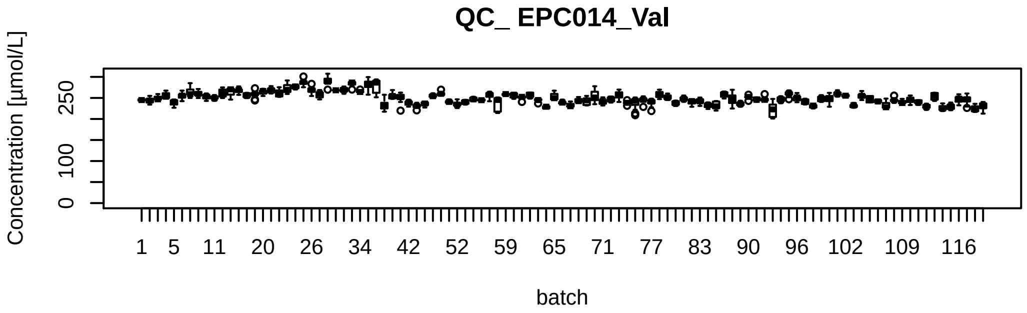
QC_ EPC014_Val
Concentration [µmol/L]
0 100 250
1 5 11 20 26 34 42 52 59 65 71 77 83 90 96 102 109 116
batch

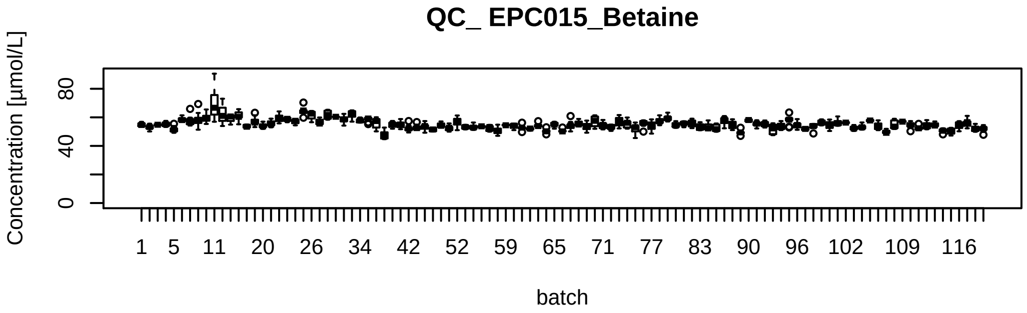
QC_ EPC015_Betaine
Concentration [µmol/L]
0 40 80
1 5 11 20 26 34 42 52 59 65 71 77 83 90 96 102 109 116
batch

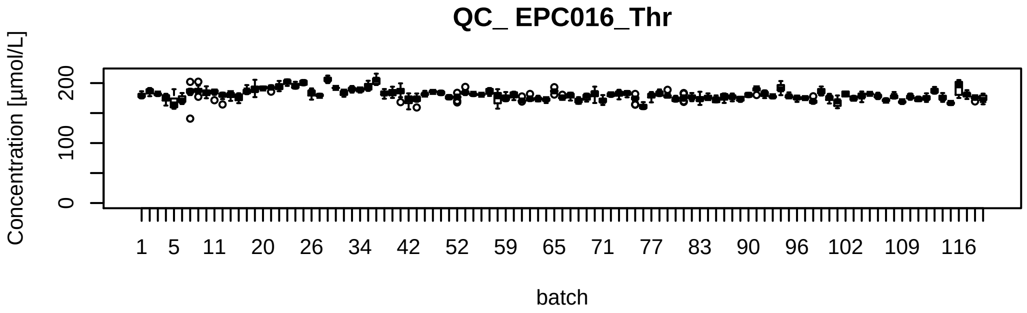
QC_ EPC016_Thr
Concentration [µmol/L]
0 100 200
1 5 11 20 26 34 42 52 59 65 71 77 83 90 96 102 109 116
batch

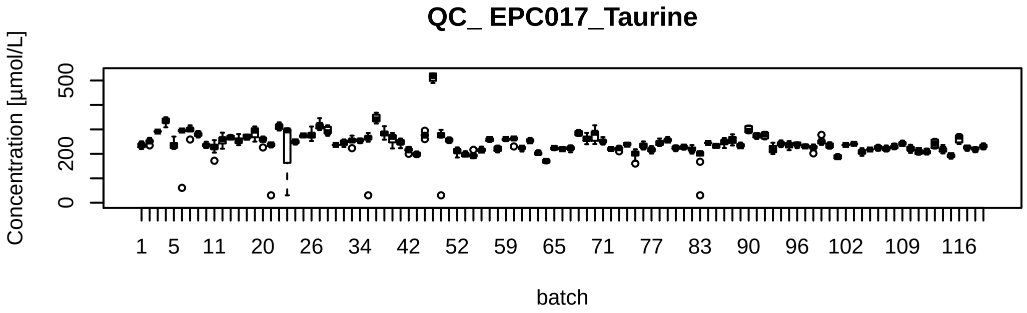
QC_ EPC017_Taurine
Concentration [µmol/L]
0
200
500
1 5 11 20 26 34 42 52 59 65 71 77 83 90 96 102 109 116
batch

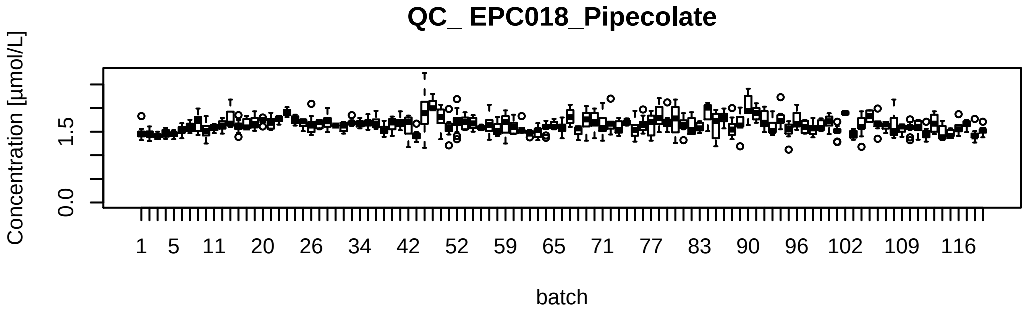
QC_ EPC018_Pipecolate
Concentration [µmol/L]
0.0
1.5
1 5 11 20 26 34 42 52 59 65 71 77 83 90 96 102 109 116
batch

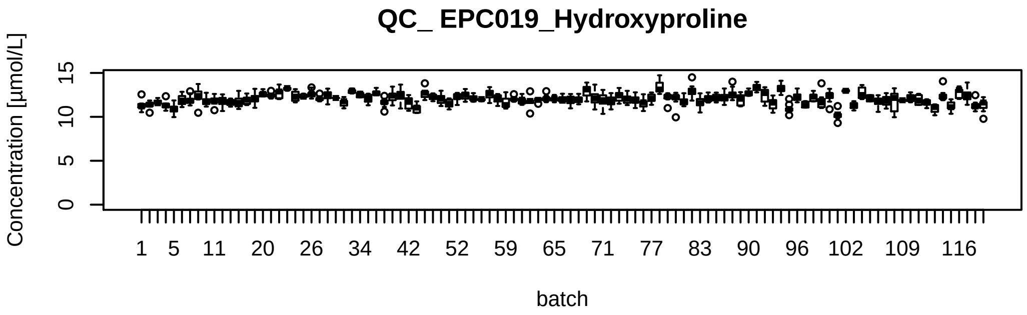
QC_ EPC019_Hydroxyproline
Concentration [µmol/L]
0
5
10
15
1 5 11 20 26 34 42 52 59 65 71 77 83 90 96 102 109 116
batch

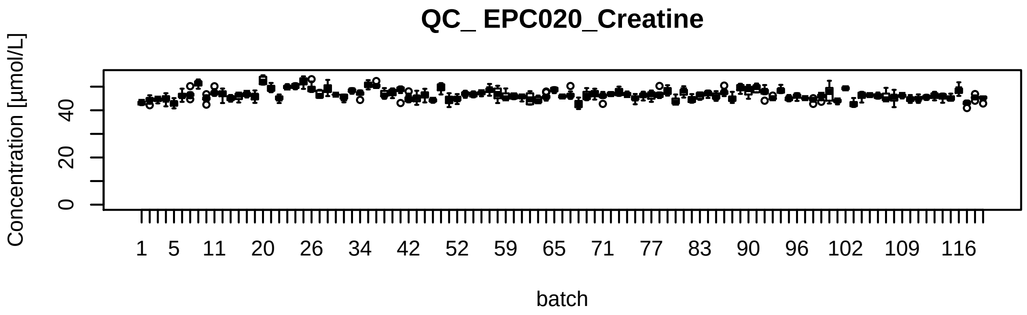
QC_ EPC020_Creatine
Concentration [µmol/L]
0
20
40
1 5 11 20 26 34 42 52 59 65 71 77 83 90 96 102 109 116
batch

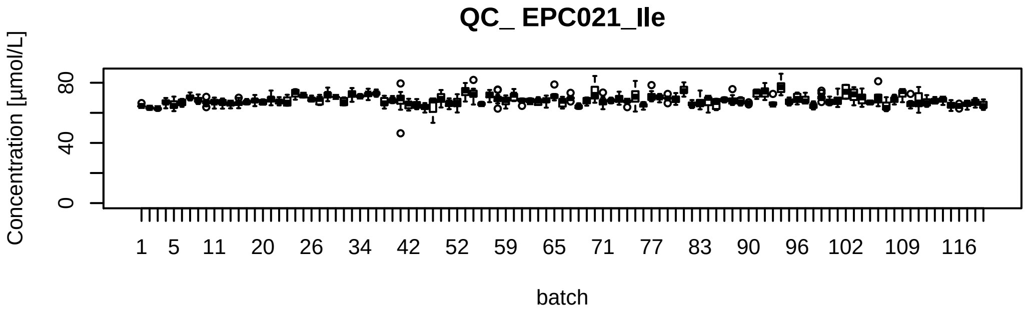
QC_ EPC021_Ile
Concentration [µmol/L]
0
40
80
1 5 11 20 26 34 42 52 59 65 71 77 83 90 96 102 109 116
batch

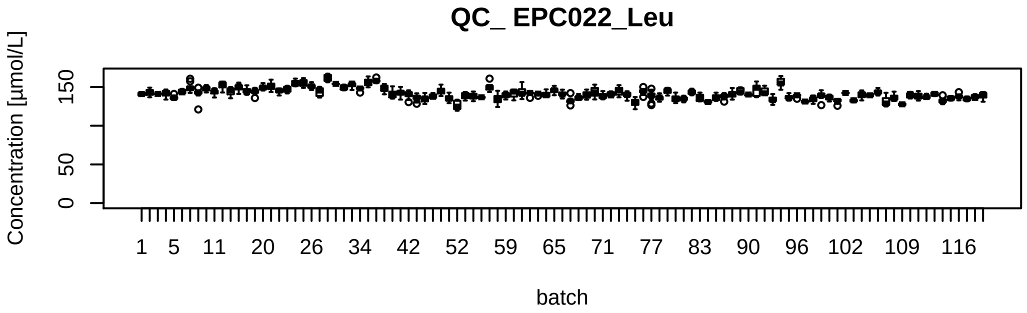
QC_ EPC022_Leu
Concentration [µmol/L]
0
50
150
1 5 11 20 26 34 42 52 59 65 71 77 83 90 96 102 109 116
batch

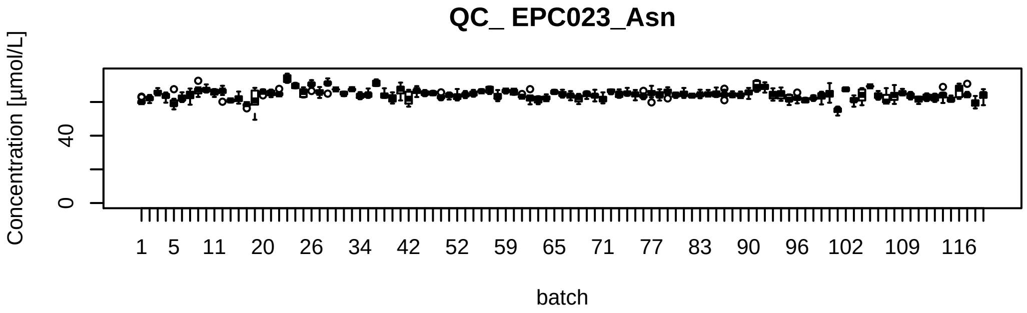
QC_ EPC023_Asn
Concentration [µmol/L]
0
40
1 5 11 20 26 34 42 52 59 65 71 77 83 90 96 102 109 116
batch

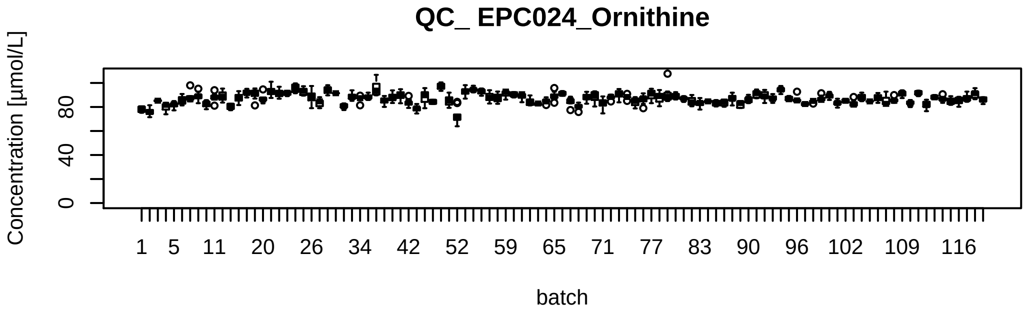
QC_ EPC024_Ornithine
Concentration [µmol/L]
0
40
80
1 5 11 20 26 34 42 52 59 65 71 77 83 90 96 102 109 116
batch

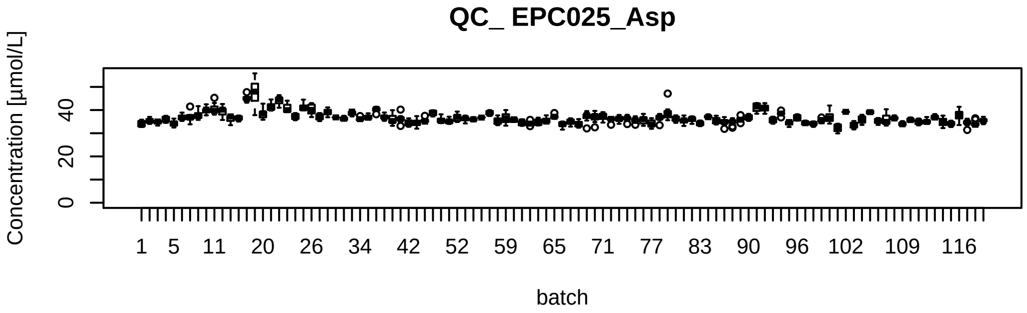
QC_ EPC025_Asp
Concentration [µmol/L]
0
20
40
1 5 11 20 26 34 42 52 59 65 71 77 83 90 96 102 109 116
batch

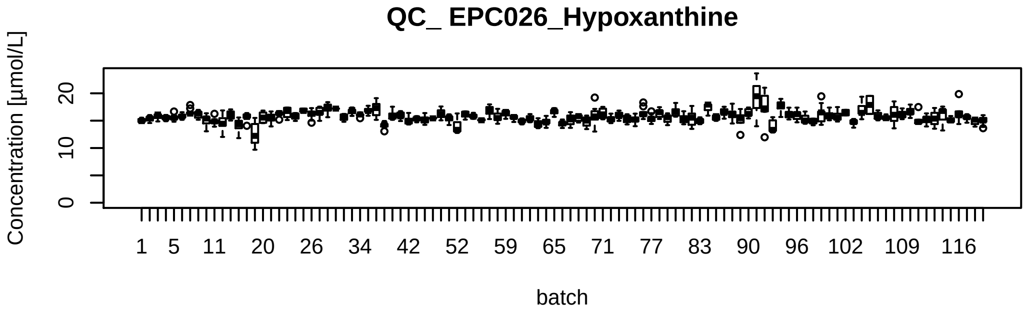
QC_ EPC026_Hypoxanthine
Concentration [µmol/L]
0
10
20
1 5 11 20 26 34 42 52 59 65 71 77 83 90 96 102 109 116
batch

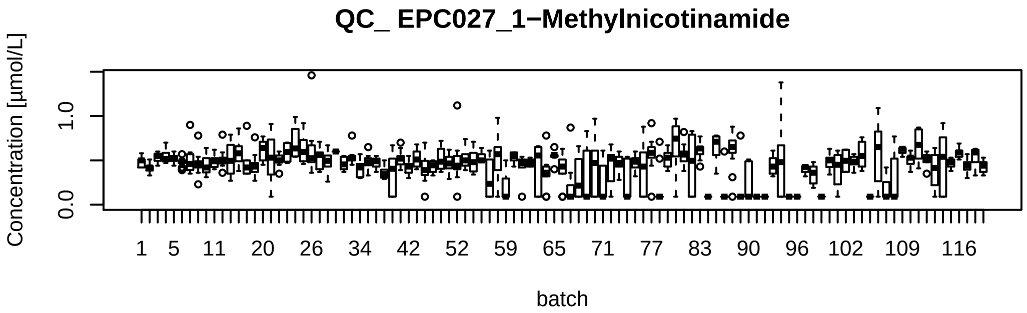
QC_ EPC027_1−Methylnicotinamide
Concentration [µmol/L]
0.0
1.0
1 5 11 20 26 34 42 52 59 65 71 77 83 90 96 102 109 116
batch

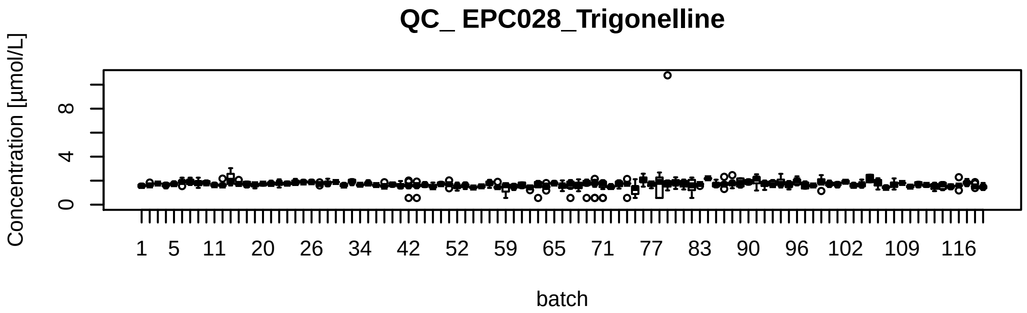
QC_ EPC028_Trigonelline
Concentration [µmol/L]
0
4
8
1 5 11 20 26 34 42 52 59 65 71 77 83 90 96 102 109 116
batch

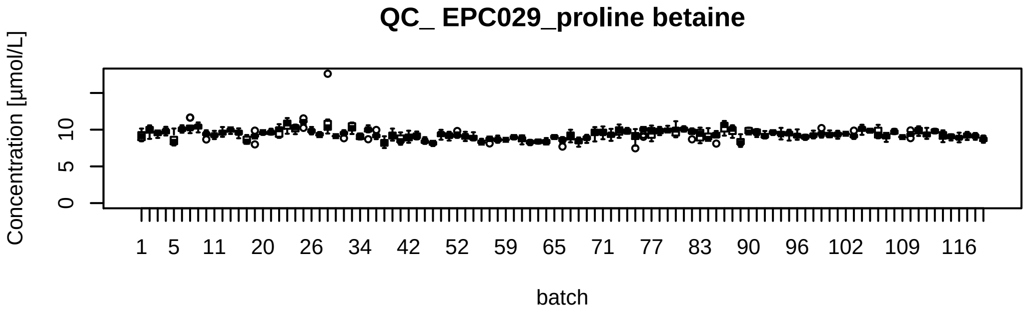
QC_ EPC029_proline betaine
Concentration [µmol/L]
0
5
10
1 5 11 20 26 34 42 52 59 65 71 77 83 90 96 102 109 116
batch

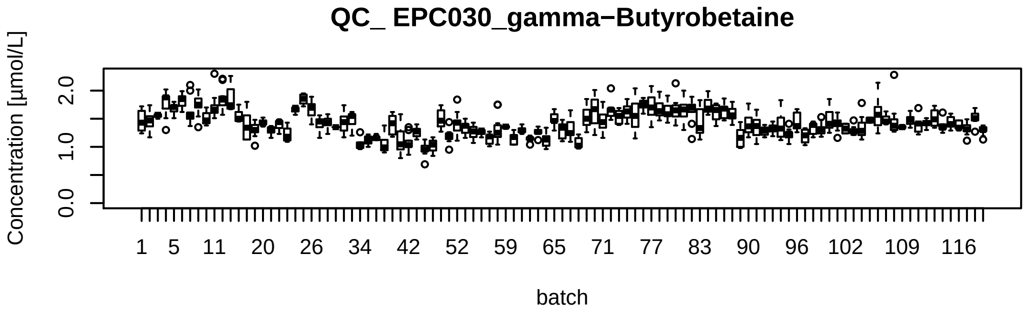
QC_ EPC030_gamma−Butyrobetaine
Concentration [µmol/L]
0.0
1.0
2.0
1 5 11 20 26 34 42 52 59 65 71 77 83 90 96 102 109 116
batch

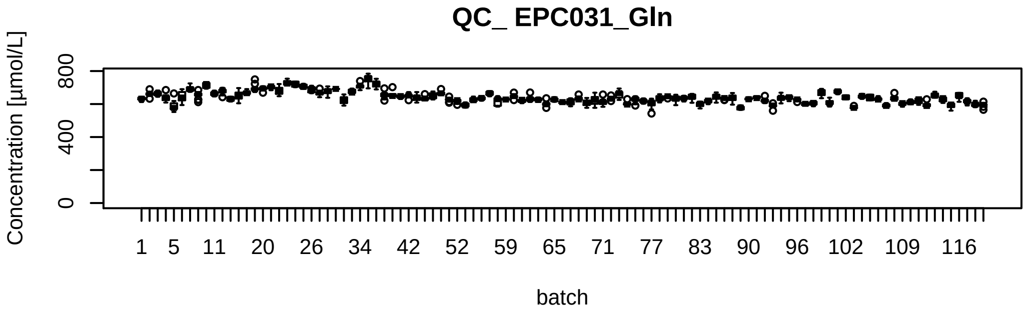
QC_ EPC031_Gln
Concentration [µmol/L]
0
400
800
1 5 11 20 26 34 42 52 59 65 71 77 83 90 96 102 109 116
batch

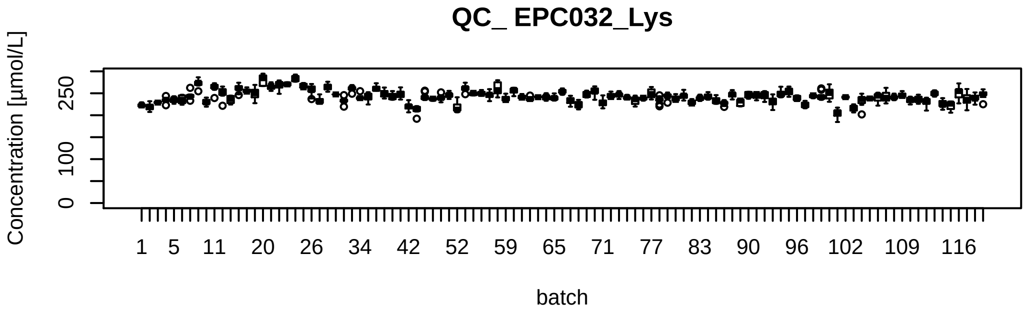
QC_ EPC032_Lys
Concentration [µmol/L]
0
100
250
1 5 11 20 26 34 42 52 59 65 71 77 83 90 96 102 109 116
batch

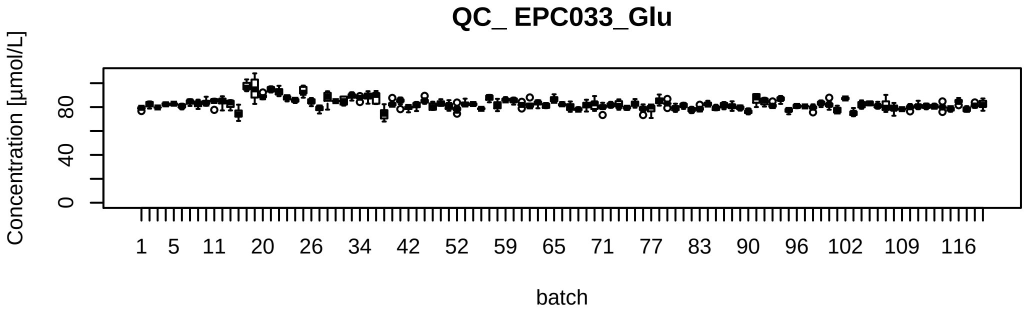
QC_ EPC033_Glu
Concentration [µmol/L]
0 40 80
1 5 11 20 26 34 42 52 59 65 71 77 83 90 96 102 109 116
batch

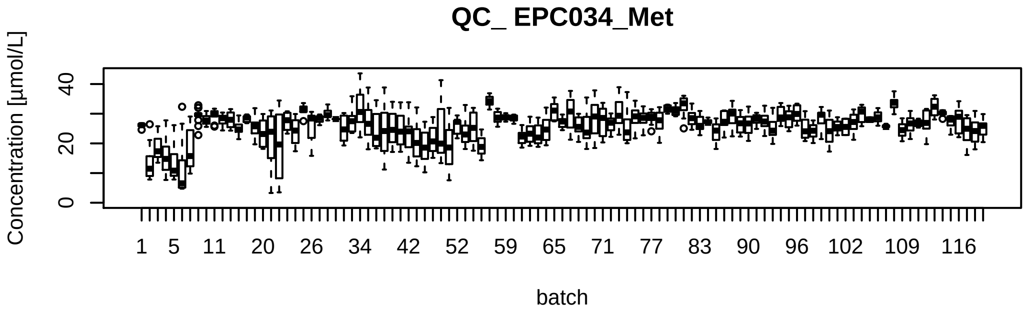
QC_ EPC034_Met
Concentration [µmol/L]
0 20 40
1 5 11 20 26 34 42 52 59 65 71 77 83 90 96 102 109 116
batch

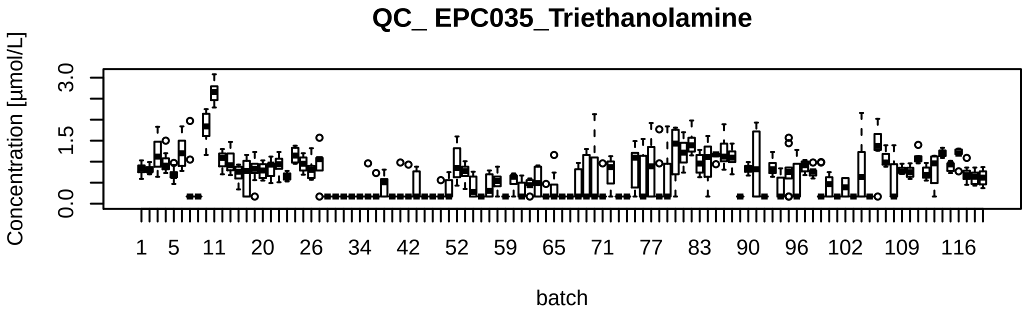
QC_ EPC035_Triethanolamine
Concentration [µmol/L]
0.0 1.5 3.0
1 5 11 20 26 34 42 52 59 65 71 77 83 90 96 102 109 116
batch

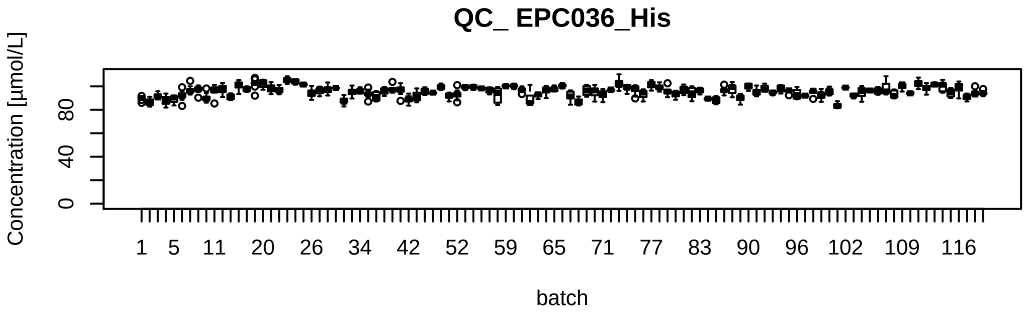
QC_ EPC036_His
Concentration [µmol/L]
0 40 80
1 5 11 20 26 34 42 52 59 65 71 77 83 90 96 102 109 116
batch

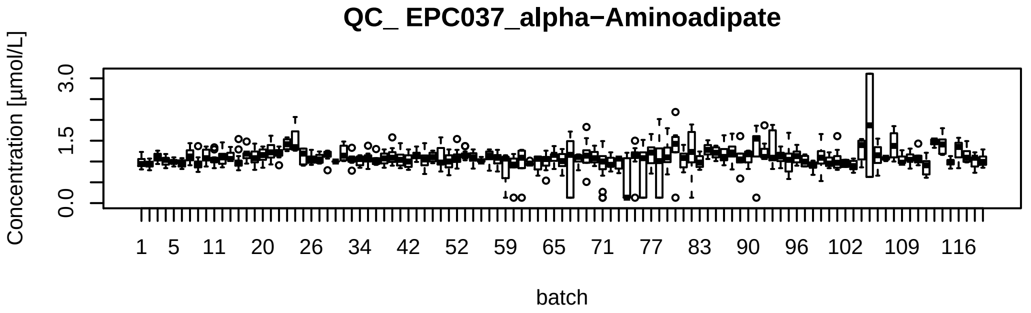
QC_ EPC037_alpha−Aminoadipate
Concentration [µmol/L]
0.0 1.5 3.0
1 5 11 20 26 34 42 52 59 65 71 77 83 90 96 102 109 116
batch

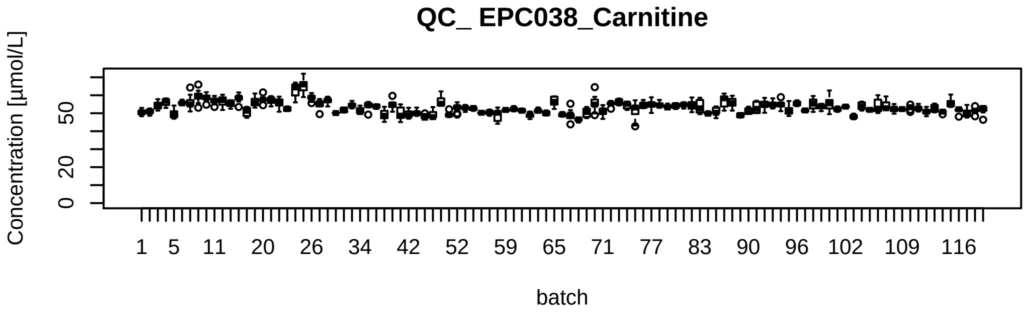
QC_ EPC038_Carnitine
Concentration [µmol/L]
0 20 50
1 5 11 20 26 34 42 52 59 65 71 77 83 90 96 102 109 116
batch

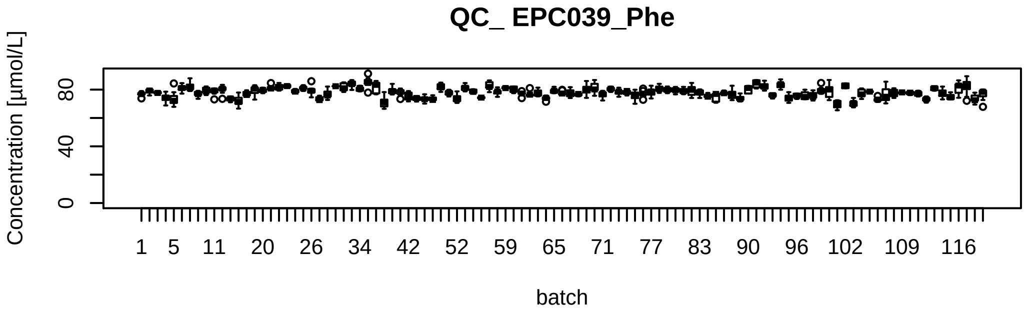
QC_ EPC039_Phe
Concentration [µmol/L]
0 40 80
1 5 11 20 26 34 42 52 59 65 71 77 83 90 96 102 109 116
batch

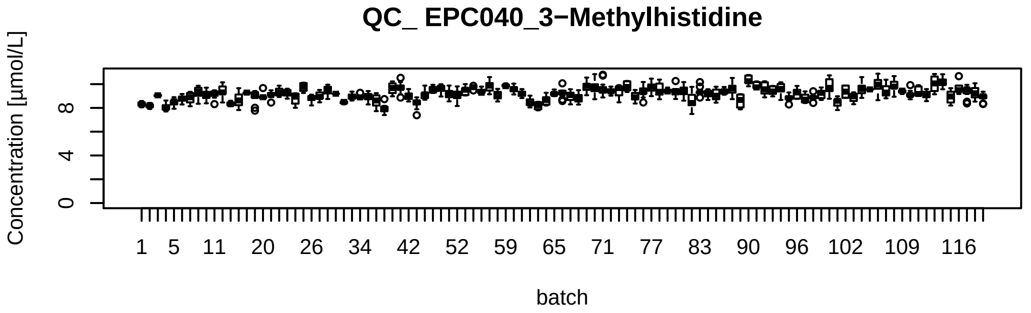
QC_ EPC040_3−Methylhistidine
Concentration [µmol/L]
0 4 8
1 5 11 20 26 34 42 52 59 65 71 77 83 90 96 102 109 116
batch

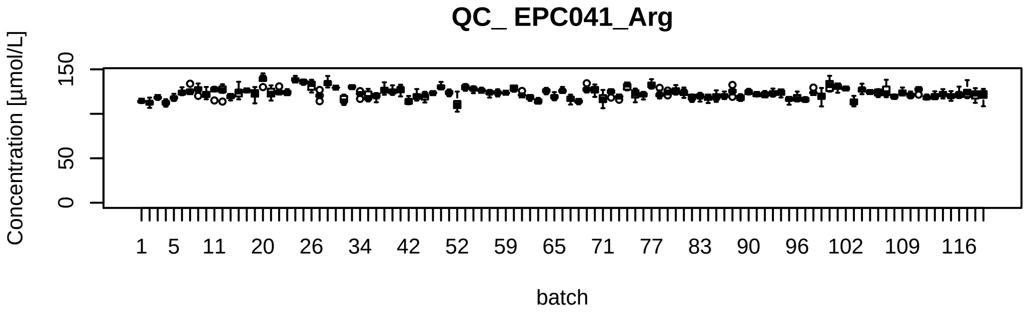
QC_ EPC041_Arg
Concentration [µmol/L]
0
50
150
1 5 11 20 26 34 42 52 59 65 71 77 83 90 96 102 109 116
batch

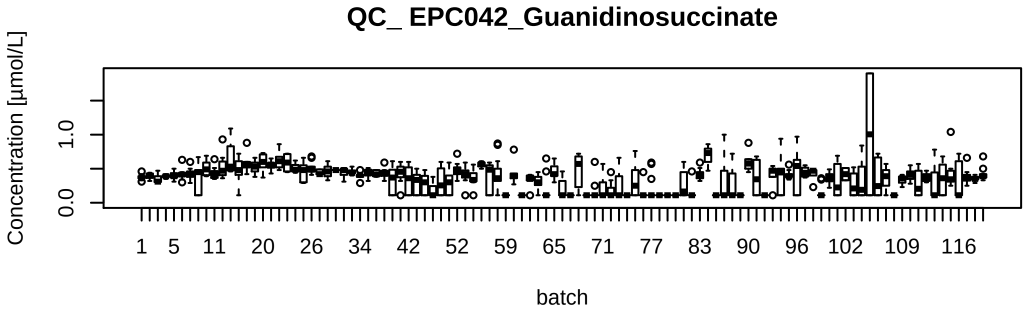
QC_ EPC042_Guanidinosuccinate
Concentration [µmol/L]
0.0
0.5
1.0
1 5 11 20 26 34 42 52 59 65 71 77 83 90 96 102 109 116
batch

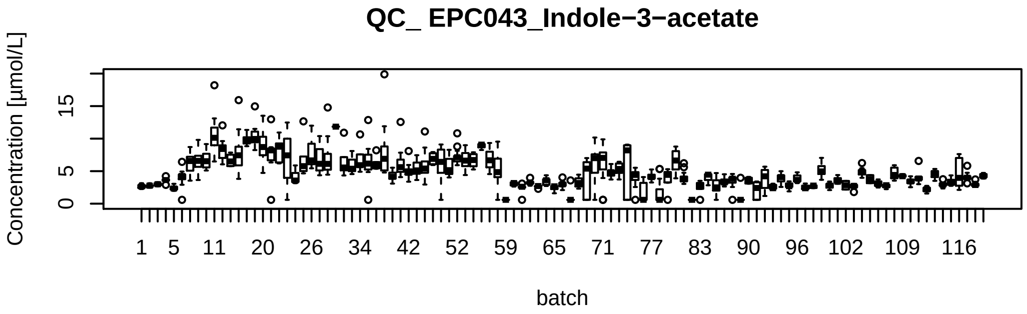
QC_ EPC043_Indole−3−acetate
Concentration [µmol/L]
0
5
15
1 5 11 20 26 34 42 52 59 65 71 77 83 90 96 102 109 116
batch

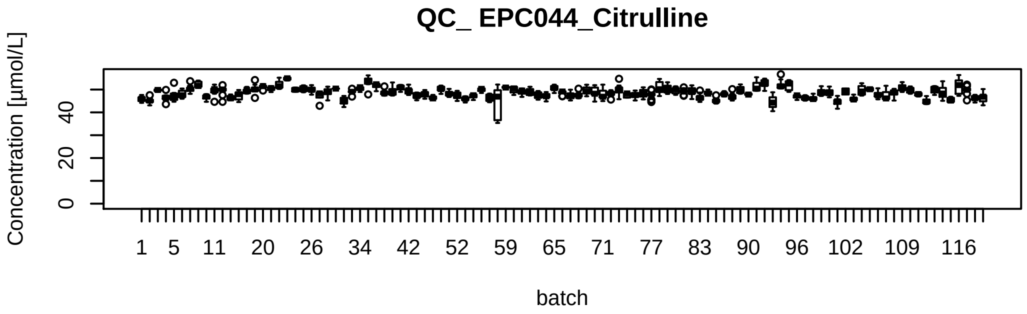
QC_ EPC044_Citrulline
Concentration [µmol/L]
0
20
40
1 5 11 20 26 34 42 52 59 65 71 77 83 90 96 102 109 116
batch

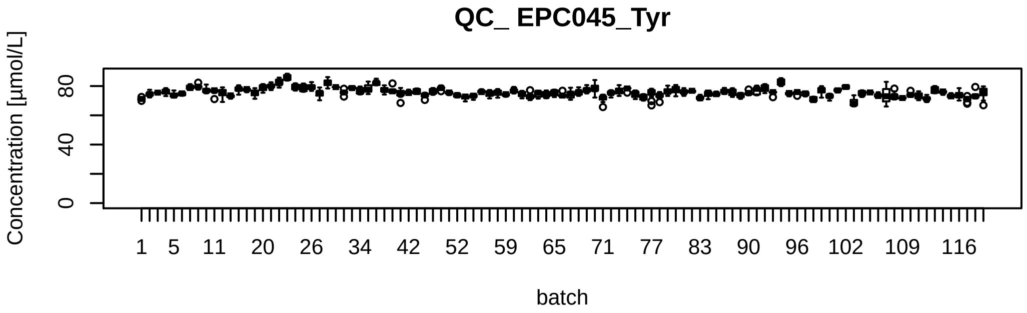
QC_ EPC045_Tyr
Concentration [µmol/L]
0
40
80
1 5 11 20 26 34 42 52 59 65 71 77 83 90 96 102 109 116
batch

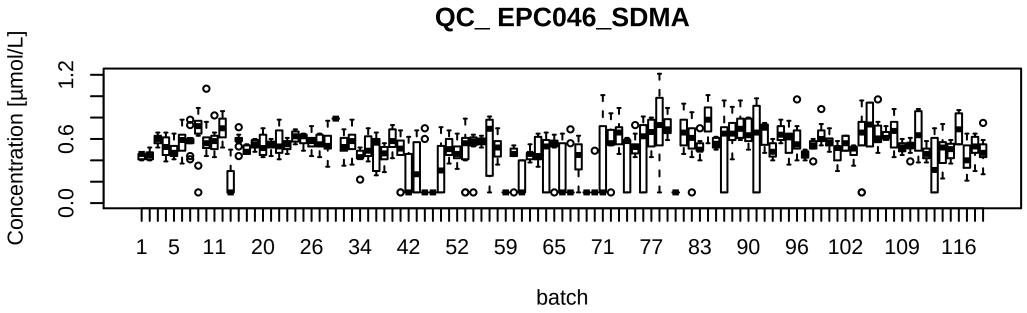
QC_ EPC046_SDMA
Concentration [µmol/L]
0.0
0.6
1.2
1 5 11 20 26 34 42 52 59 65 71 77 83 90 96 102 109 116
batch

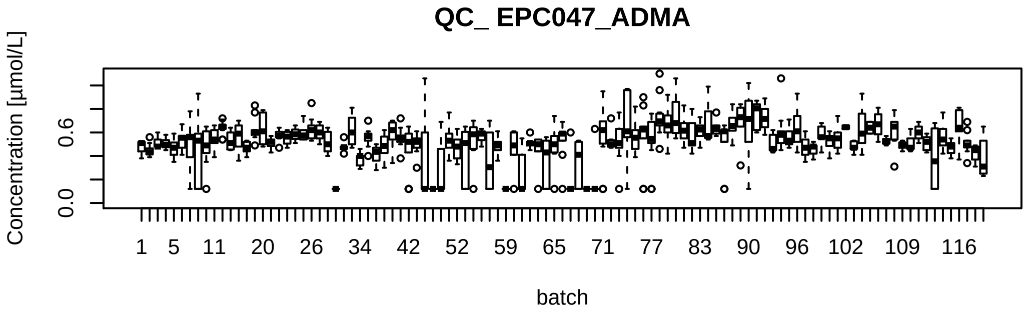
QC_ EPC047_ADMA
Concentration [µmol/L]
0.0
0.6
1 5 11 20 26 34 42 52 59 65 71 77 83 90 96 102 109 116
batch

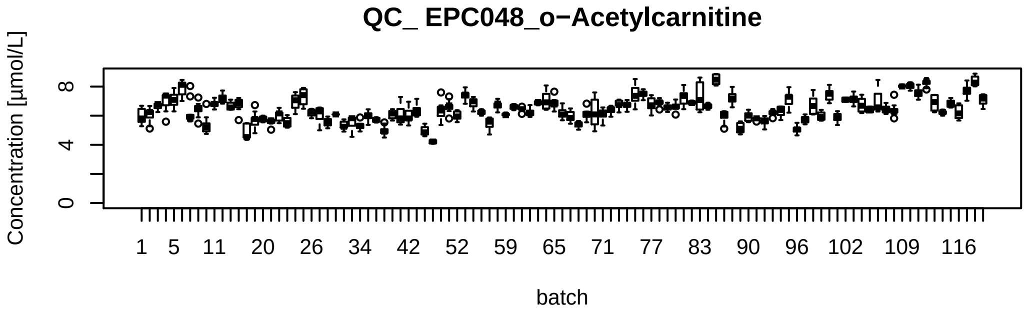
QC_ EPC048_o−Acetylcarnitine
Concentration [µmol/L]
0
4
8
1 5 11 20 26 34 42 52 59 65 71 77 83 90 96 102 109 116
batch

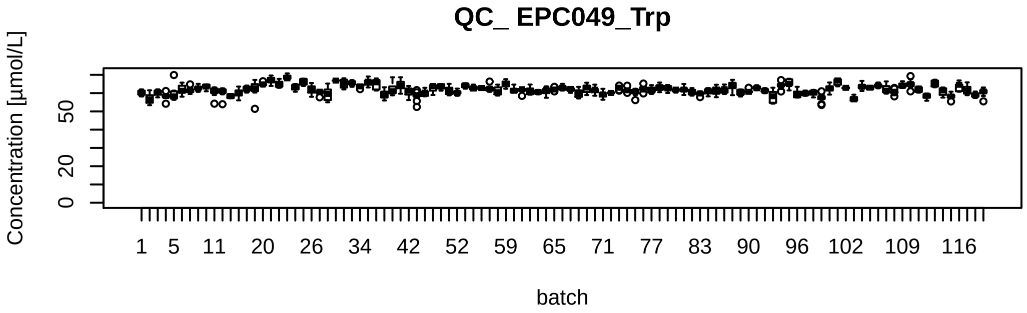
QC_ EPC049_Trp
Concentration [µmol/L]
0 20 50
1 5 11 20 26 34 42 52 59 65 71 77 83 90 96 102 109 116
batch

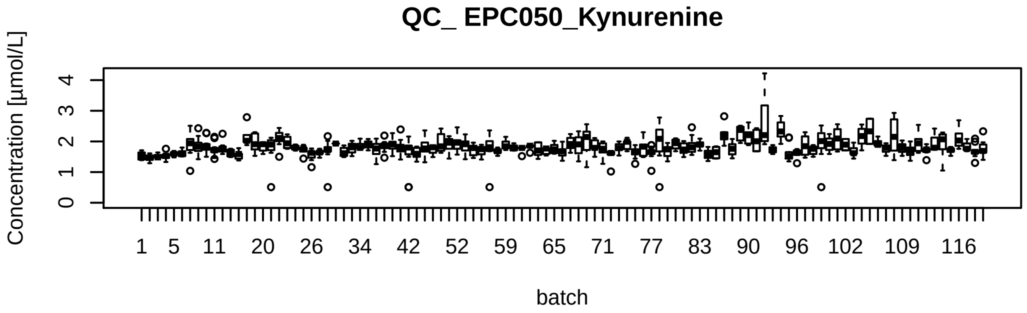
QC_ EPC050_Kynurenine
Concentration [µmol/L]
0 1 2 3 4
1 5 11 20 26 34 42 52 59 65 71 77 83 90 96 102 109 116
batch

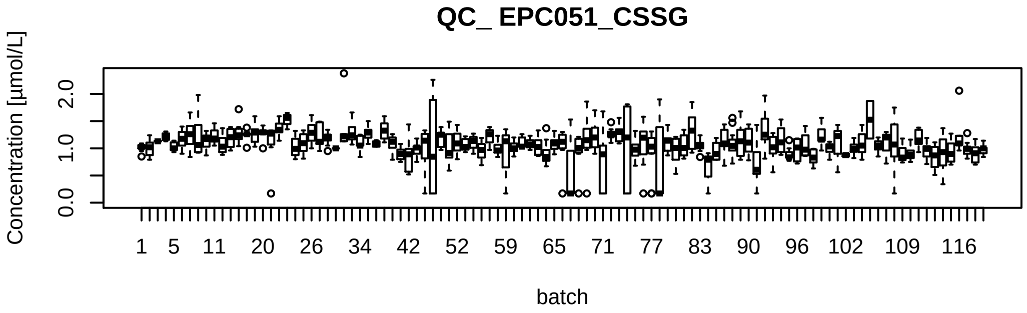
QC_ EPC051_CSSG
Concentration [µmol/L]
0.0 1.0 2.0
1 5 11 20 26 34 42 52 59 65 71 77 83 90 96 102 109 116
batch

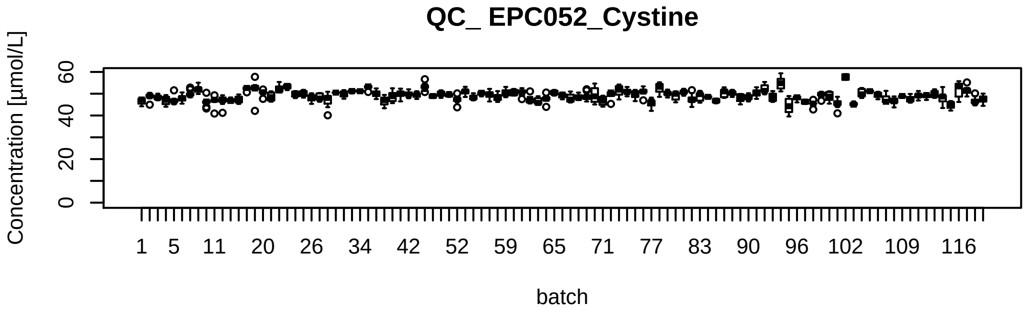
QC_ EPC052_Cystine
Concentration [µmol/L]
0 20 40 60
1 5 11 20 26 34 42 52 59 65 71 77 83 90 96 102 109 116
batch

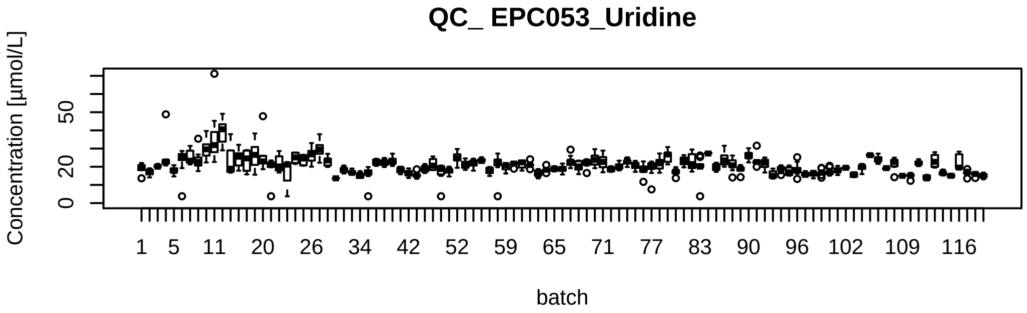
QC_ EPC053_Uridine
Concentration [µmol/L]
0 20 50
1 5 11 20 26 34 42 52 59 65 71 77 83 90 96 102 109 116
batch

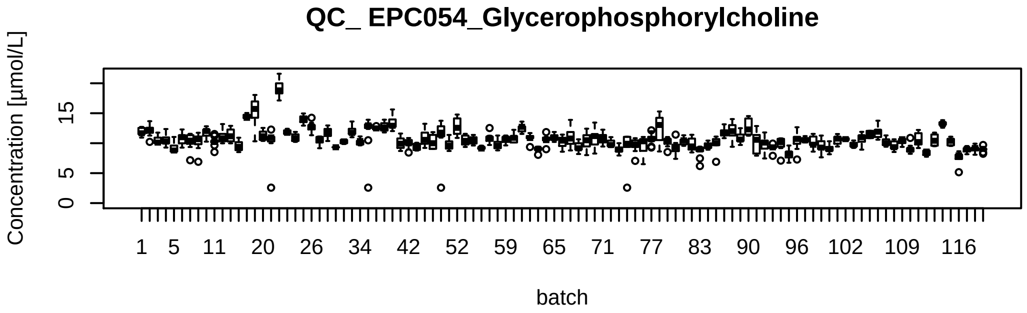
QC_ EPC054_Glycerophosphorylcholine
Concentration [µmol/L]
0 5 15
1 5 11 20 26 34 42 52 59 65 71 77 83 90 96 102 109 116
batch
