# Supplementary material for: Reliability of plasma polar metabolite concentrations in a large-scale cohort study using capillary electrophoresis-mass spectrometry
Source: PLoS One. 2018 Jan 18;13(1):e0191230. doi: 10.1371/journal.pone.0191230 (PMC5773198; doi:10.1371/journal.pone.0191230)
Supplement: S2 File — (PDF) [file pone.0191230.s003.pdf]

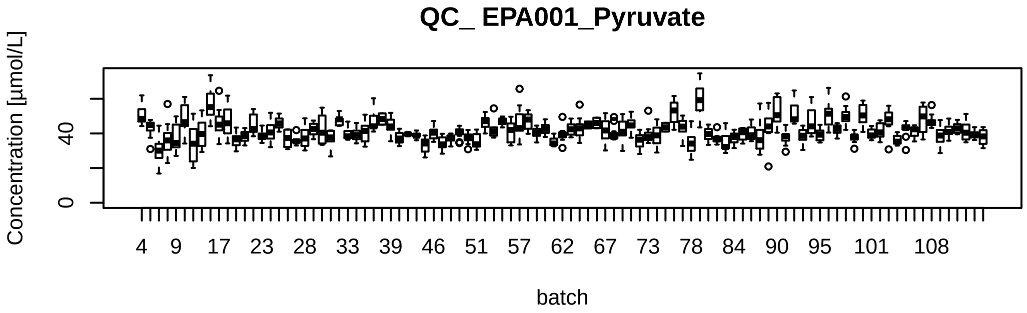
QC_ EPA001_Pyruvate
Concentration [µmol/L]
0
40
4 9 17 23 28 33 39 46 51 57 62 67 73 78 84 90 95 101 108
batch
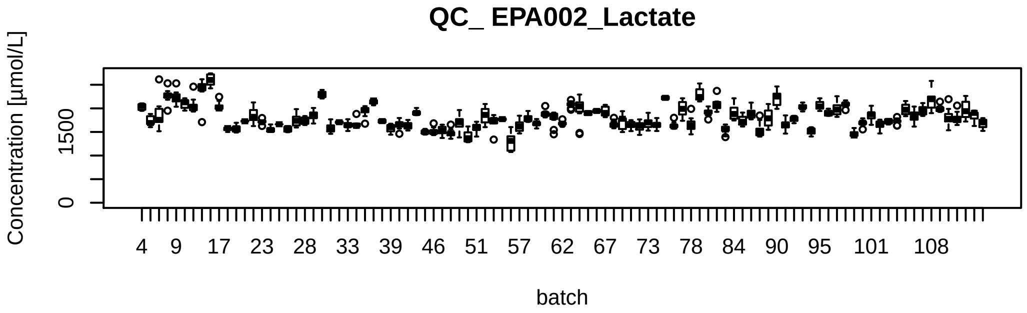
QC_ EPA002_Lactate
Concentration [µmol/L]
0
1500
4 9 17 23 28 33 39 46 51 57 62 67 73 78 84 90 95 101 108
batch
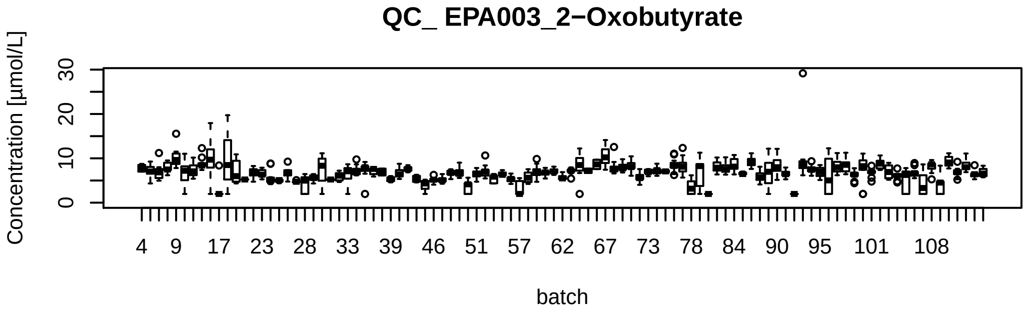
QC_ EPA003_2−Oxobutyrate
Concentration [µmol/L]
0
10
20
30
4 9 17 23 28 33 39 46 51 57 62 67 73 78 84 90 95 101 108
batch
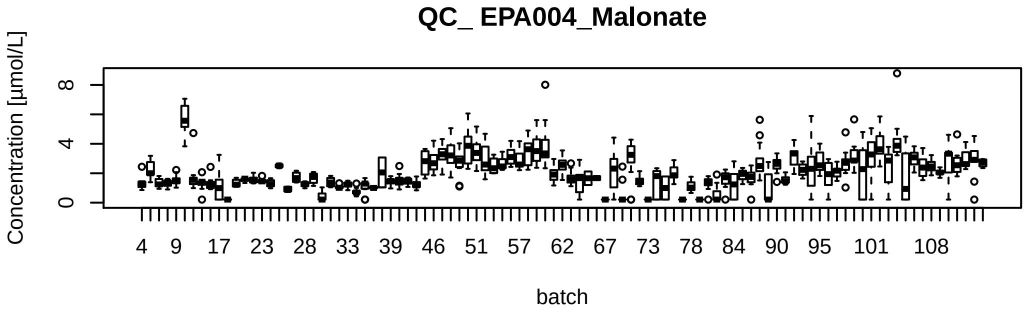
QC_ EPA004_Malonate
Concentration [µmol/L]
0
4
8
4 9 17 23 28 33 39 46 51 57 62 67 73 78 84 90 95 101 108
batch
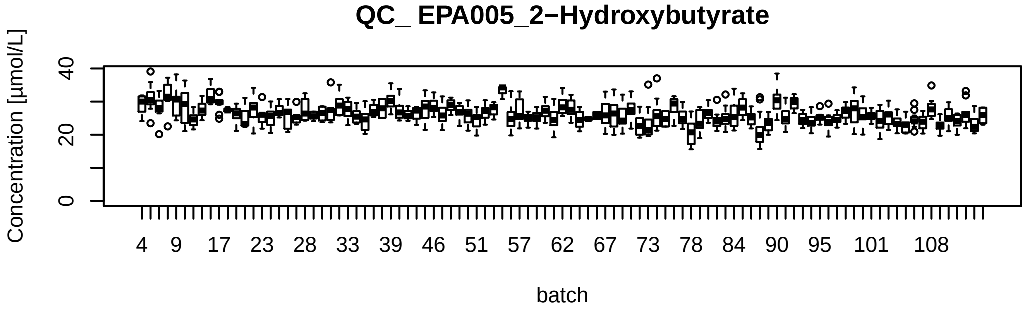
QC_ EPA005_2−Hydroxybutyrate
Concentration [µmol/L]
0
20
40
4 9 17 23 28 33 39 46 51 57 62 67 73 78 84 90 95 101 108
batch
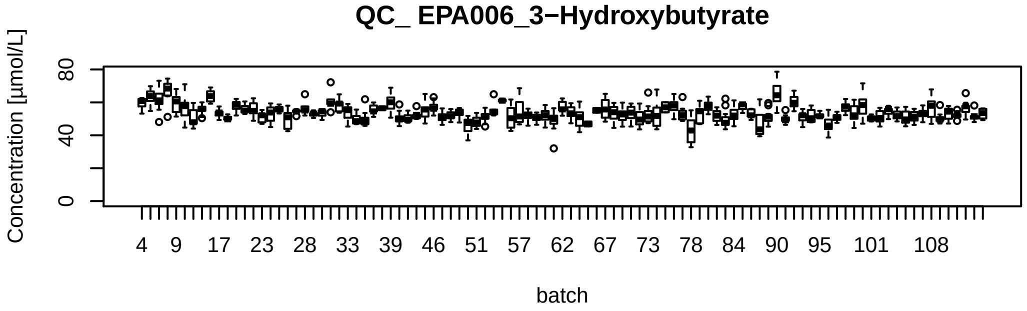
QC_ EPA006_3−Hydroxybutyrate
Concentration [µmol/L]
0
40
80
4 9 17 23 28 33 39 46 51 57 62 67 73 78 84 90 95 101 108
batch
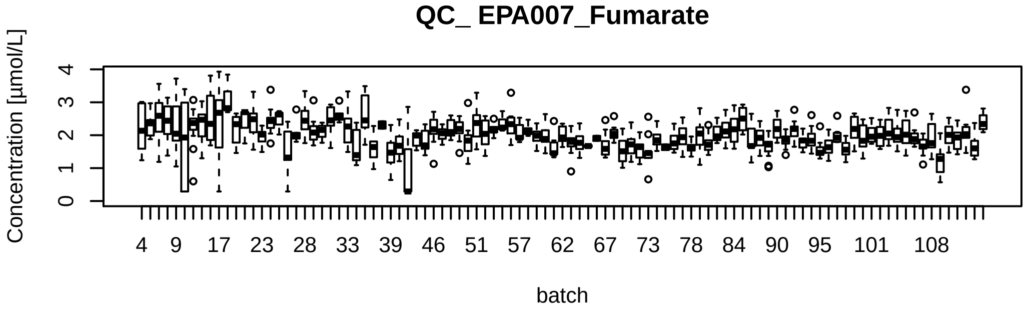
QC_ EPA007_Fumarate
Concentration [µmol/L]
0
1
2
3
4
4 9 17 23 28 33 39 46 51 57 62 67 73 78 84 90 95 101 108
batch
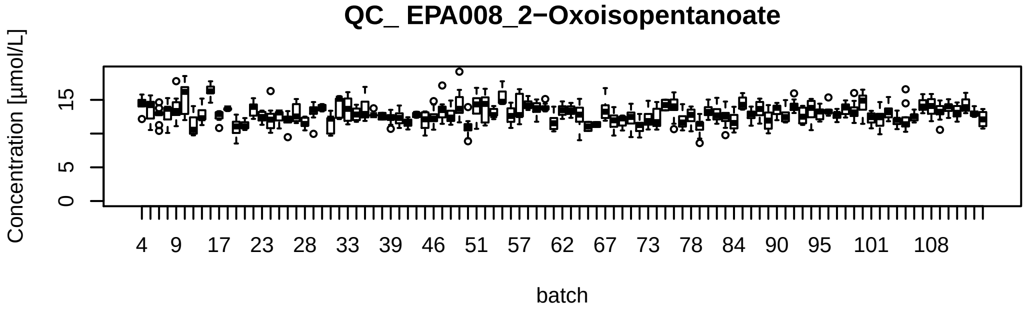
QC_ EPA008_2−Oxoisopentanoate
Concentration [µmol/L]
0
5
15
4 9 17 23 28 33 39 46 51 57 62 67 73 78 84 90 95 101 108
batch

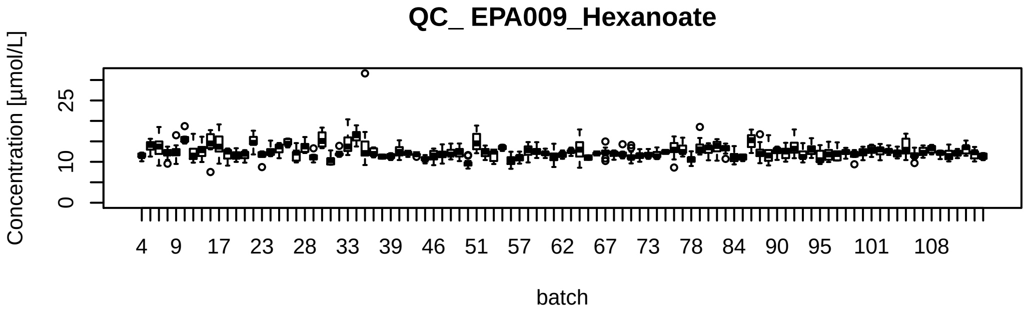
QC_ EPA009_Hexanoate
Concentration [µmol/L]
0
10
25
4 9 17 23 28 33 39 46 51 57 62 67 73 78 84 90 95 101 108
batch

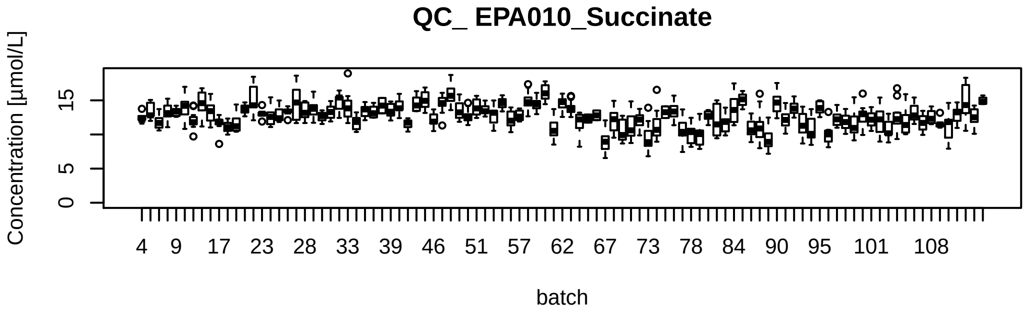
QC_ EPA010_Succinate
Concentration [µmol/L]
0
5
10
15
4 9 17 23 28 33 39 46 51 57 62 67 73 78 84 90 95 101 108
batch

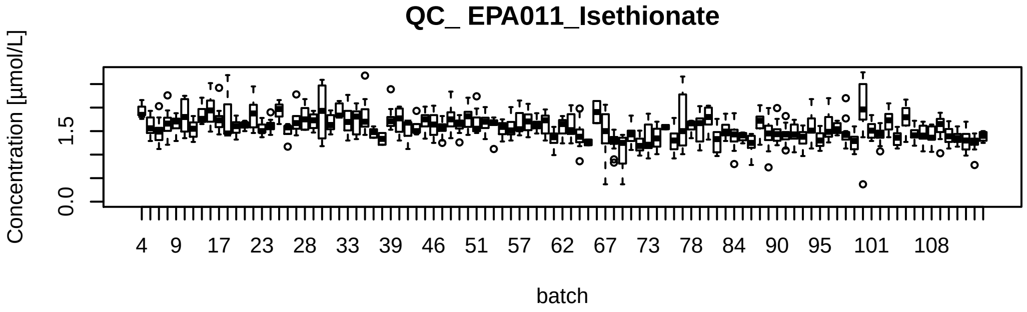
QC_ EPA011_Isethionate
Concentration [µmol/L]
0.0
1.5
4 9 17 23 28 33 39 46 51 57 62 67 73 78 84 90 95 101 108
batch

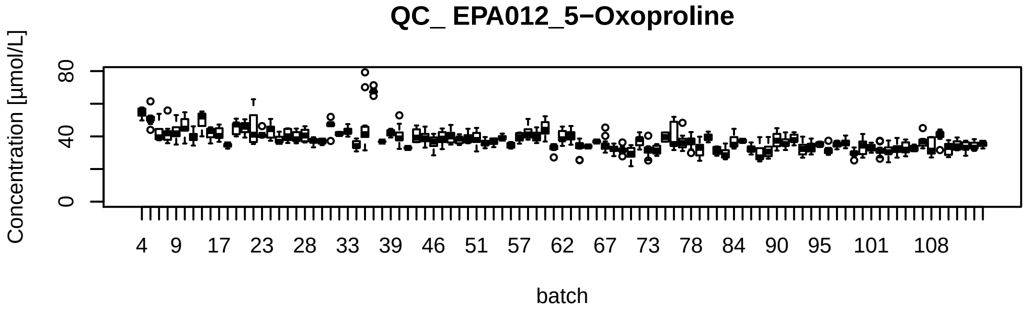
QC_ EPA012_5−Oxoproline
Concentration [µmol/L]
0
40
80
4 9 17 23 28 33 39 46 51 57 62 67 73 78 84 90 95 101 108
batch

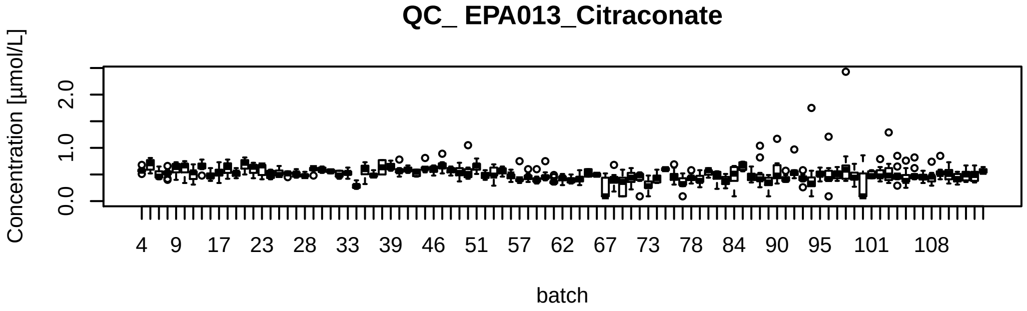
QC_ EPA013_Citraconate
Concentration [µmol/L]
0.0
1.0
2.0
4 9 17 23 28 33 39 46 51 57 62 67 73 78 84 90 95 101 108
batch

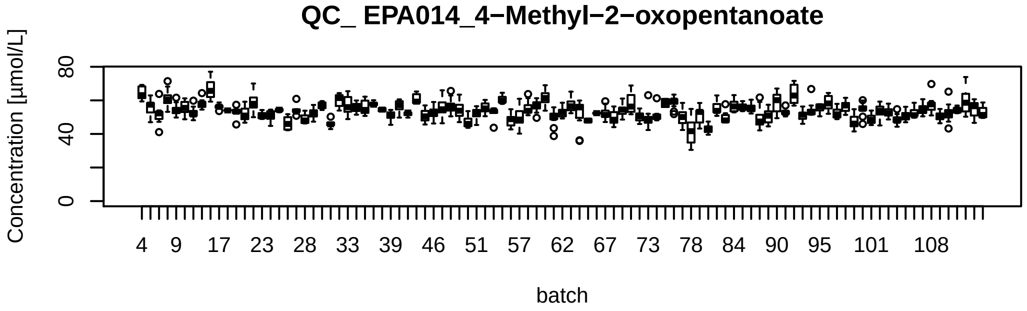
QC_ EPA014_4−Methyl−2−oxopentanoate
Concentration [µmol/L]
0
40
80
4 9 17 23 28 33 39 46 51 57 62 67 73 78 84 90 95 101 108
batch

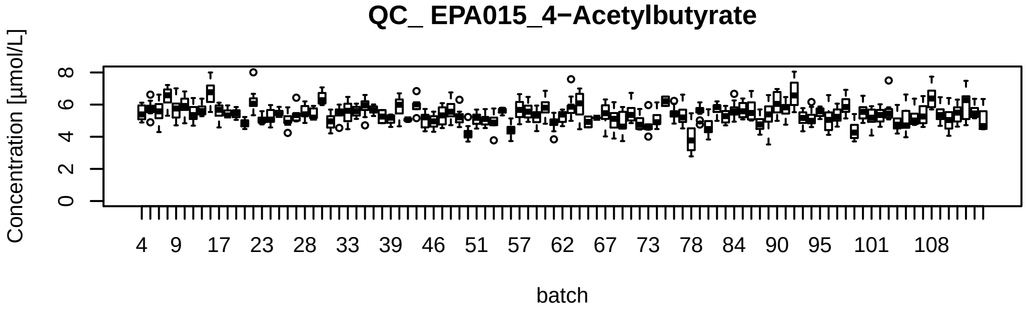
QC_ EPA015_4−Acetylbutyrate
Concentration [µmol/L]
0
2
4
6
8
4 9 17 23 28 33 39 46 51 57 62 67 73 78 84 90 95 101 108
batch

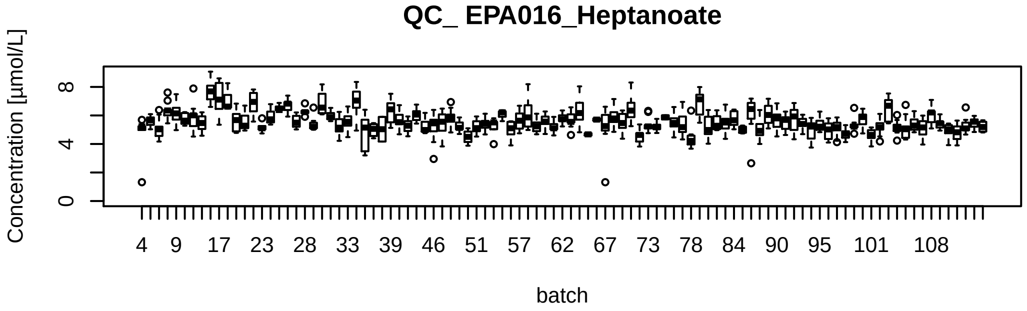
QC_ EPA016_Heptanoate
Concentration [µmol/L]
0
4
8
4 9 17 23 28 33 39 46 51 57 62 67 73 78 84 90 95 101 108
batch

QC_ EPA017_Glutarate

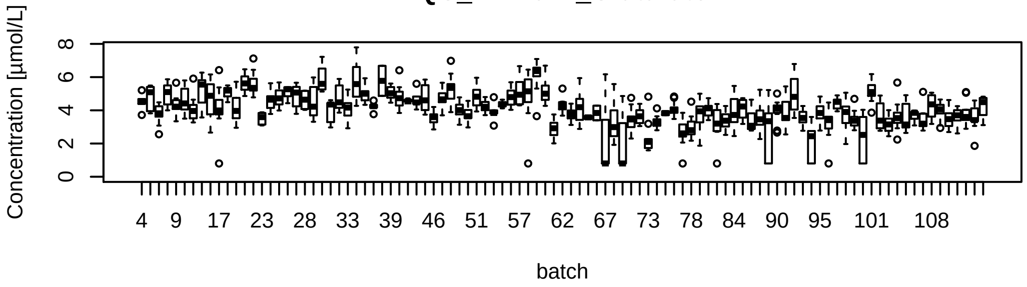

QC_ EPA018_Malate

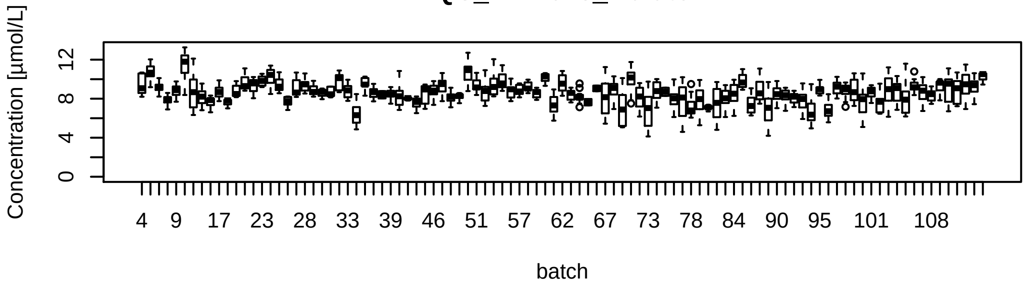

QC_ EPA019_threonate

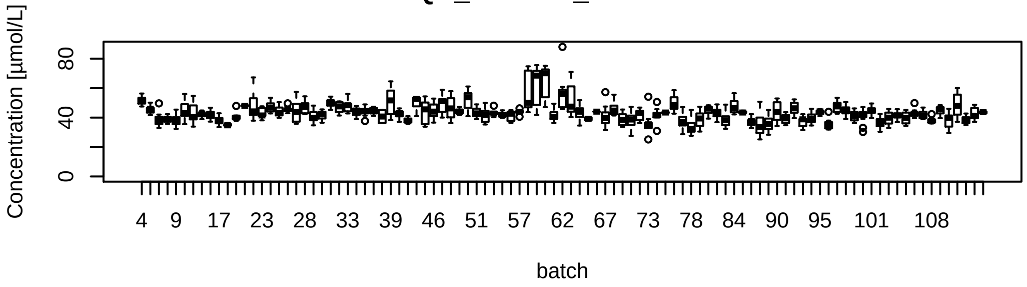

QC_ EPA020_Ethanolamine phosphate

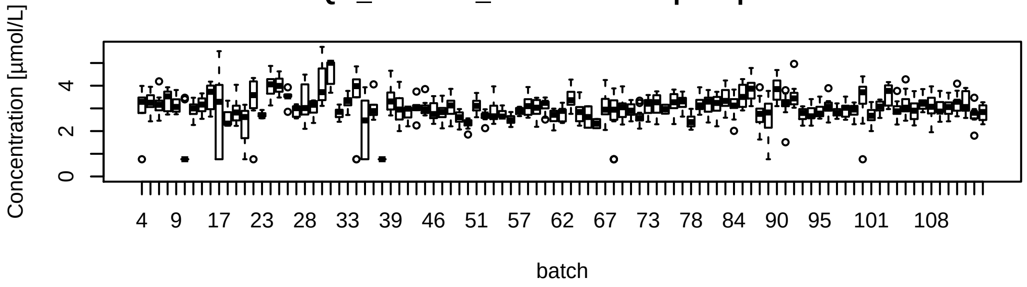

QC_ EPA021_Octanoate

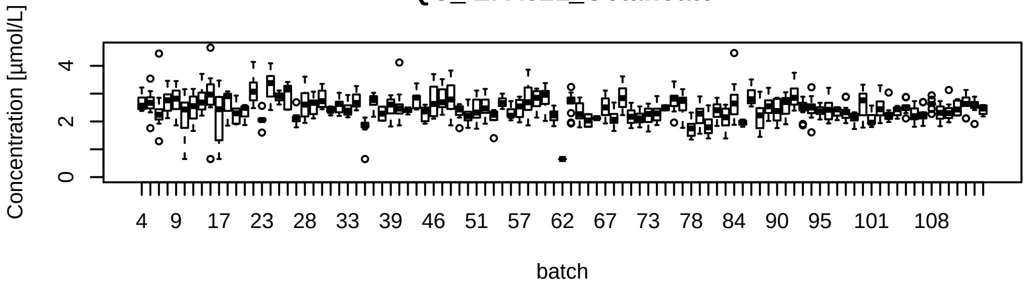

QC_ EPA022_2−Oxoglutarate

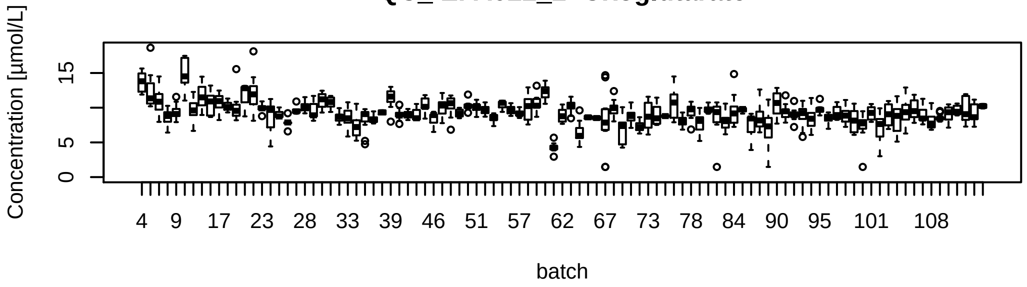

QC_ EPA023_Pelargonate

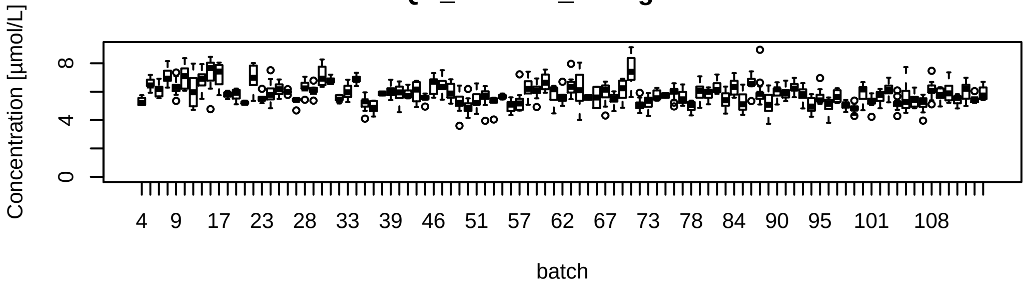

QC_ EPA024_Terephthalate

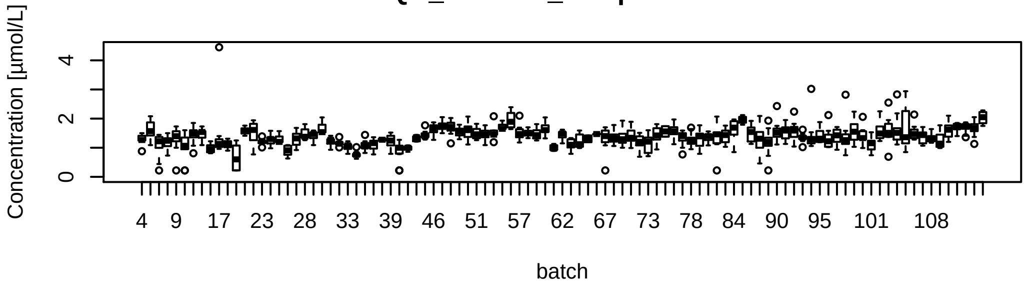

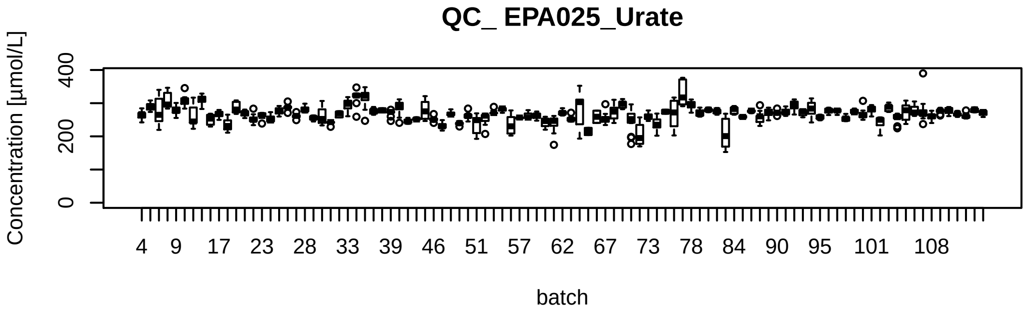
QC_ EPA025_Urate
Concentration [µmol/L]
0
200
400
4 9 17 23 28 33 39 46 51 57 62 67 73 78 84 90 95 101 108
batch

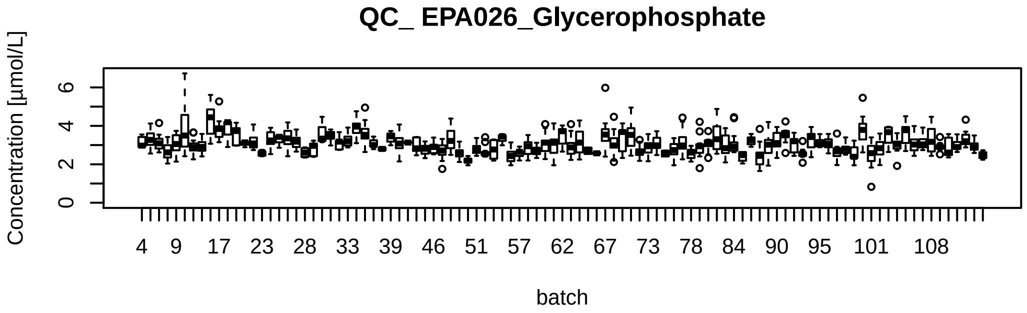
QC_ EPA026_Glycerophosphate
Concentration [µmol/L]
0
2
4
6
4 9 17 23 28 33 39 46 51 57 62 67 73 78 84 90 95 101 108
batch

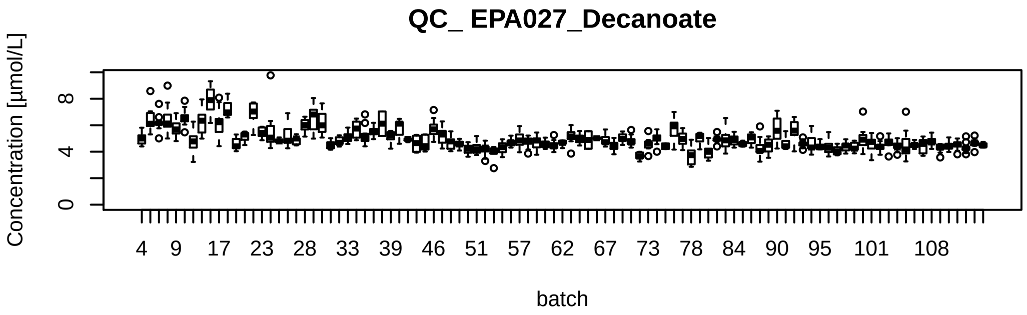
QC_ EPA027_Decanoate
Concentration [µmol/L]
0
4
8
4 9 17 23 28 33 39 46 51 57 62 67 73 78 84 90 95 101 108
batch

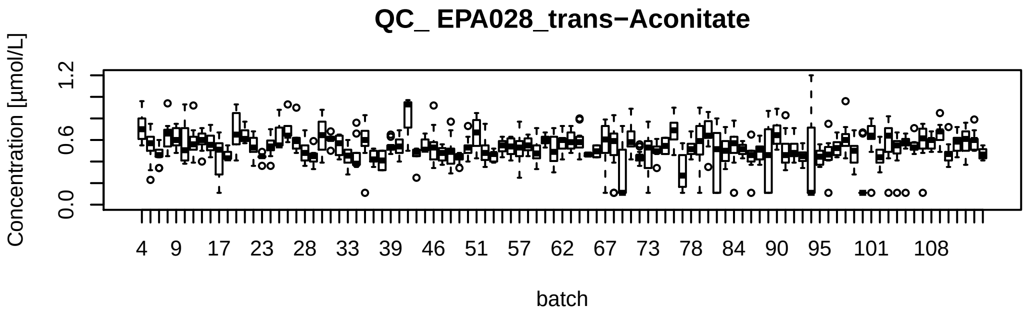
QC_ EPA028_trans−Aconitate
Concentration [µmol/L]
0.0
0.6
1.2
4 9 17 23 28 33 39 46 51 57 62 67 73 78 84 90 95 101 108
batch

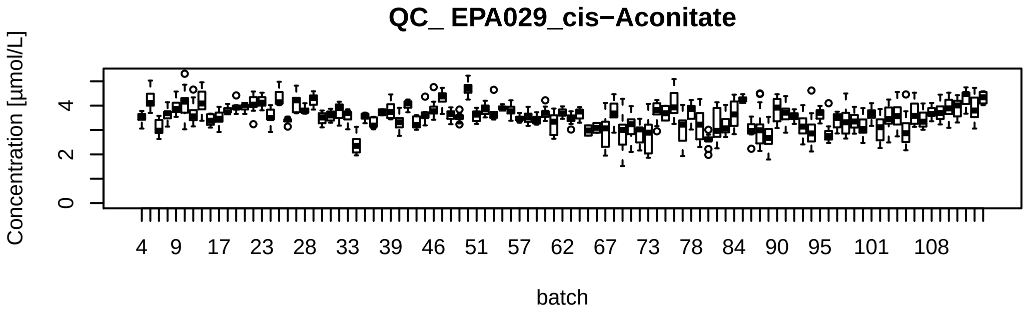
QC_ EPA029_cis−Aconitate
Concentration [µmol/L]
0
2
4
4 9 17 23 28 33 39 46 51 57 62 67 73 78 84 90 95 101 108
batch

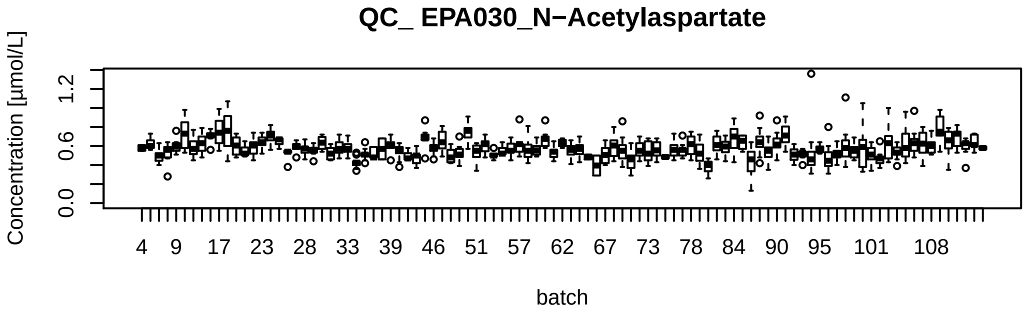
QC_ EPA030_N−Acetylaspartate
Concentration [µmol/L]
0.0
0.6
1.2
4 9 17 23 28 33 39 46 51 57 62 67 73 78 84 90 95 101 108
batch

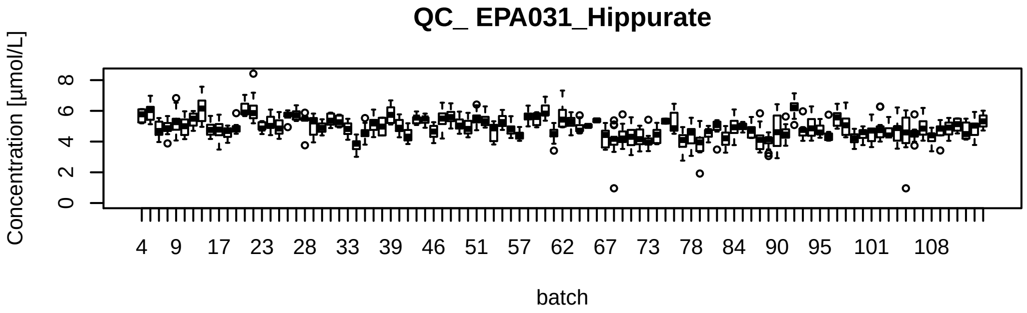
QC_ EPA031_Hippurate
Concentration [µmol/L]
0
2
4
6
8
4 9 17 23 28 33 39 46 51 57 62 67 73 78 84 90 95 101 108
batch

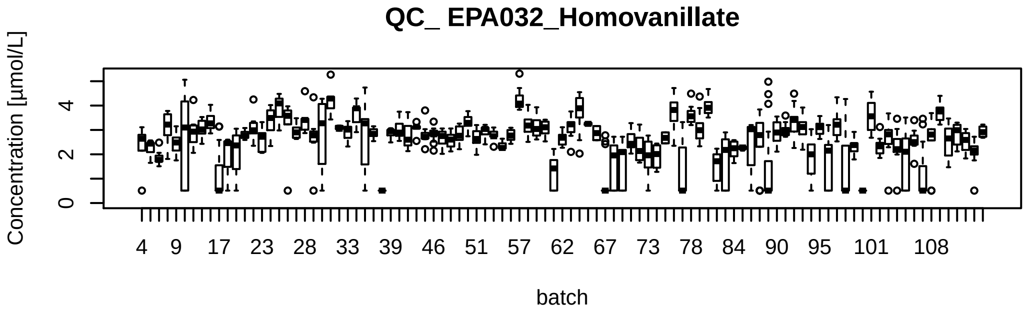
QC_ EPA032_Homovanillate
Concentration [µmol/L]
0
2
4
4 9 17 23 28 33 39 46 51 57 62 67 73 78 84 90 95 101 108
batch

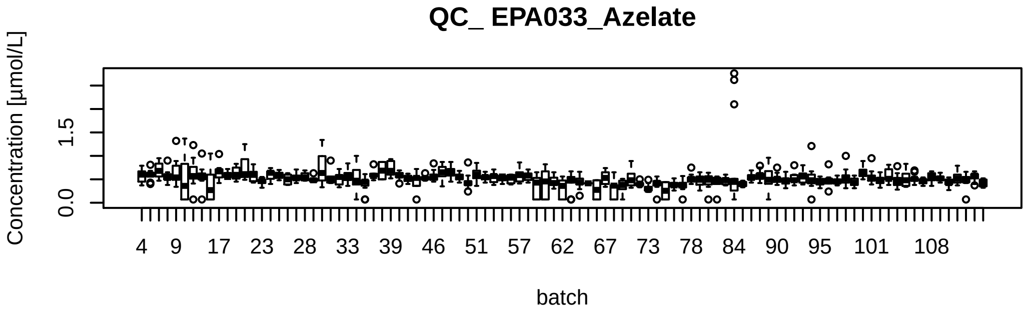
QC_ EPA033_Azelate
Concentration [µmol/L]
0.0
1.5
4 9 17 23 28 33 39 46 51 57 62 67 73 78 84 90 95 101 108
batch

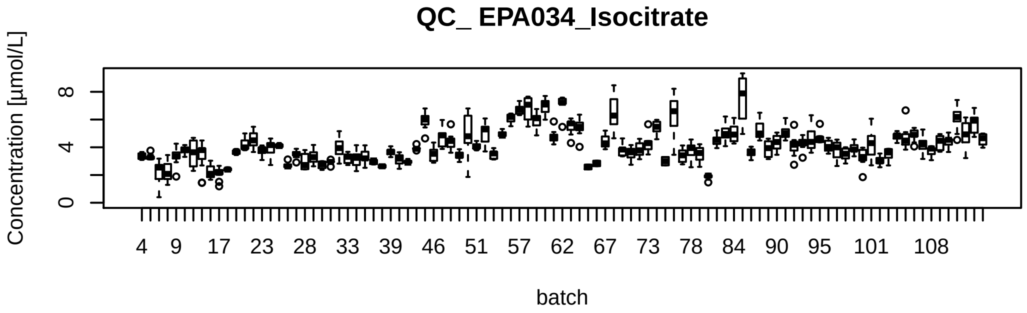
QC_ EPA034_Isocitrate
Concentration [µmol/L]
0
4
8
4 9 17 23 28 33 39 46 51 57 62 67 73 78 84 90 95 101 108
batch

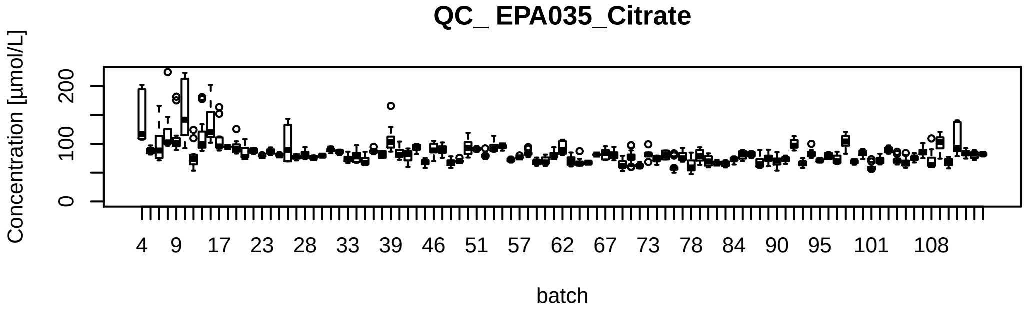
QC_ EPA035_Citrate
Concentration [µmol/L]
0
100
200
4 9 17 23 28 33 39 46 51 57 62 67 73 78 84 90 95 101 108
batch

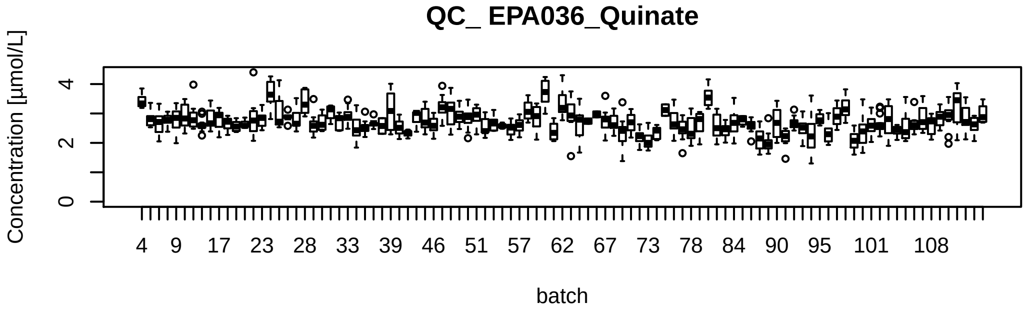
QC_ EPA036_Quinate
Concentration [µmol/L]
0
2
4
4 9 17 23 28 33 39 46 51 57 62 67 73 78 84 90 95 101 108
batch

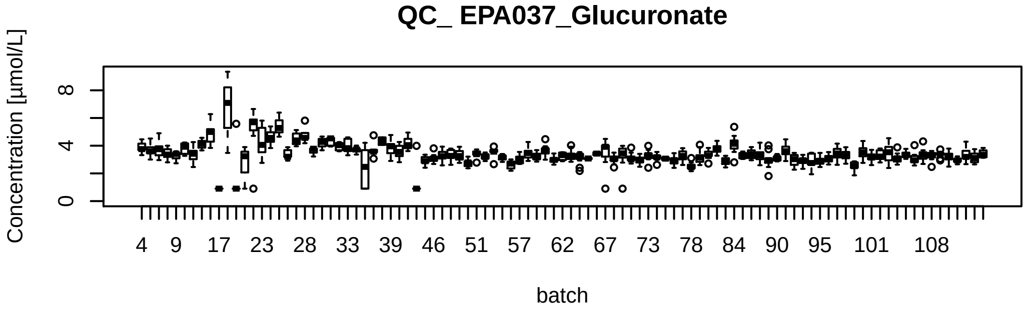
QC_ EPA037_Glucuronate
Concentration [µmol/L]
0
4
8
4 9 17 23 28 33 39 46 51 57 62 67 73 78 84 90 95 101 108
batch

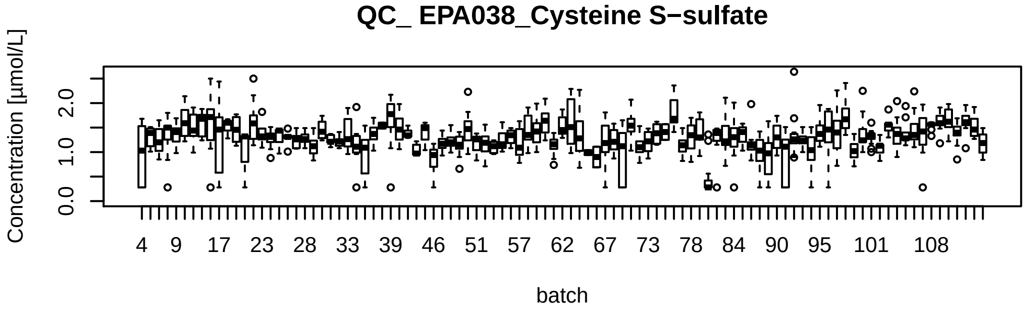
QC_ EPA038_Cysteine S−sulfate
Concentration [µmol/L]
0.0
1.0
2.0
4 9 17 23 28 33 39 46 51 57 62 67 73 78 84 90 95 101 108
batch

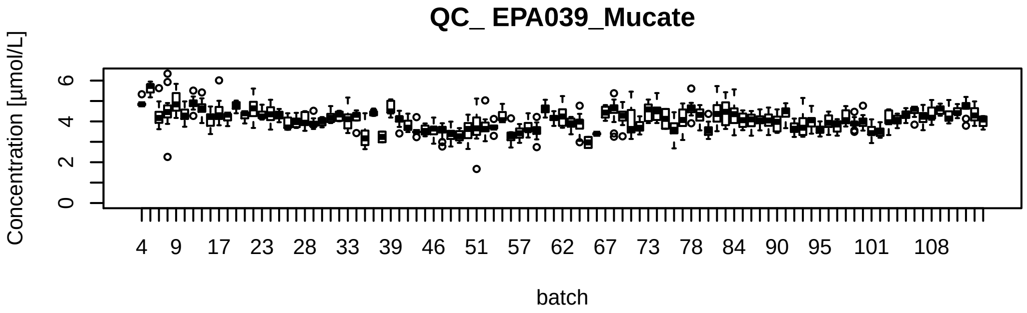
QC_ EPA039_Mucate
Concentration [µmol/L]
0
2
4
6
4 9 17 23 28 33 39 46 51 57 62 67 73 78 84 90 95 101 108
batch

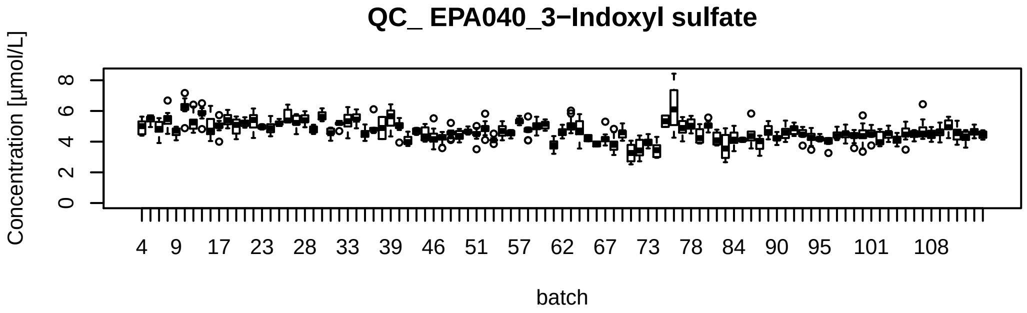
QC_ EPA040_3−Indoxyl sulfate
Concentration [µmol/L]
0
2
4
6
8
4 9 17 23 28 33 39 46 51 57 62 67 73 78 84 90 95 101 108
batch
